# Differentiation in Protest Politics: Participation by Political Insiders and Outsiders

**DOI:** 10.1007/s11109-022-09846-7

**Published:** 2023-01-12

**Authors:** Endre Borbáth

**Affiliations:** 1 Institute of Sociology, Freie Universität Berlin, Garystraße 55, 14195 Berlin, Germany; 2 Center for Civil Society Research, WZB Berlin Social Science Center, Reichpietschufer 50, 10785 Berlin, Germany

**Keywords:** Political participation, Covid-19, Protest, Germany, Political issues, Surveys

## Abstract

The political participation literature has documented a long-term trend of the normalization of noninstitutional participation that is often equated with the conventionalization of engagement in protest politics. Less is known on the extent to which noninstitutional forms are differentiated by their mobilization context. Population surveys find it difficult to contextualize individual engagement, and on-site surveys point to effects that are hard to generalize. This study fills this gap by emphasizing differentiation and distinguishing participation according to the issue of engagement. It introduces a conceptual distinction between political insiders and outsiders, defined based on the extent to which they are embedded in the organizational landscape of the dominant cleavage dimension. Using an original survey conducted in Germany during the Covid-19 crisis, the analysis demonstrates that general-population surveys are fit to examine issue-specific participation patterns. The results expose an insider and outsider divide, captured by the effect of attitudinal and behavioral indicators, and demonstrates that the two groups are equally likely to participate in noninstitutional forms. However, insiders engage on the established issues of climate and anti-racism, whereas outsiders engage on the new issues of Covid-19 related economic assistance and civil liberties restrictions. In addition, dynamic models reveal that noninstitutional participation is rooted in volatile issue preferences. Overall, the paper argues that participation during the Covid-19 crisis has furthered the trend towards a differentiated protest arena.

**Supplementary Information:**

The online version contains supplementary material available at 10.1007/s11109-022-09846-7.

## Introduction

In a seminal contribution published more than 40 years ago, Barnes and Kaase (1979) methodologically and substantively initiated the study of European protest with general-population surveys. They coined the term “unconventional” participation to describe various forms of participation outside of voting. However, the label “unconventional” became the most contested part of the study, particularly because participation rates were high across all the forms of protest they examined. In addition, a series of later findings demonstrated that people who protest are politically interested and tend to also vote or otherwise participate in representative channels (Aelst & Walgrave, 2001; Lahusen & Bleckmann, 2015; Norris et al., 2005; Saunders, 2014). As a result, the subsequent literature largely abandoned the label “unconventional” in favor of alternatives such as “noninstitutional” participation (e.g., Hooghe & Marien, 2013). This literature shows that, in advanced democracies, noninstitutional participation by and large became normalized, available in the participation repertoire of a great number of citizens (Borbáth & Gessler, 2020; Jeroense & Spierings, 2022; Meyer & Tarrow, 1998; Oser, 2017, 2021).

Research on the normalization of protest has mostly focused on the extent to which noninstitutional forms became conventionalized, referring to their availability in the participation repertoire of citizens with diverse socio-demographic, ideological and attitudinal backgrounds. Driven by the assumption that people who participate are more likely to see their issue preferences represented in decision-making processes, this perspective focuses on socio-demographic inequalities in participation (e.g., Dalton et al., 2010; Verba et al., 1995). Notwithstanding the importance of conventionalization, another important component of normalization refers to differentiation, namely to the idea that *“protest behavior is employed with greater frequency, by more diverse constituencies, and is used to represent a wider range of claims than ever before”* (Meyer & Tarrow, 1998, p. 4), also see: Rucht and Neidhardt 2002). However, empirical research with general-population surveys has largely abandoned the idea of individual-level differences being a function of the meso-level context of mobilization, leading (Kriesi, 2008, p. 148) to diagnose an “individualistic bias” in the study of noninstitutional participation. This is not simply a historical oversight: when they describe most of the survey items general-population surveys (still) rely on to measure noninstitutional participation, Barnes and Kaase (1979) explicitly argue that the mobilization context plays little to no role in respondents” decision to participate (p. 67).

The current paper studies the differentiation of noninstitutional forms of participation by highlighting issue-specific engagement. Asking *to what extent and how do individuals who participate on various issues differ*, the paper makes a substantive and a methodological contribution. Substantively, it introduces the distinction between political insiders and outsiders to show that both groups participate in noninstitutional forms. Methodologically, it illustrates the importance of differentiating participation by the issue context of engagement using a general-population survey. For this reason, the paper introduces a novel survey item on issues of participation that allows me to examine how individuals who participate in various meso-level mobilization contexts differ. The paper bridges two scholarly traditions: the noninstitutional participation research strand, which uses general-population survey, and the social movement studies tradition that uses on-site surveys. As the analysis demonstrates, the variation both between and within protesting “crowds” can be modeled using general-population surveys. This approach has the advantage that it avoids sampling on the dependent variable and by design includes nonparticipants, without resorting to additional samples or empirical corrections (Saunders & Shlomo, 2021).

To examine the differentiation of noninstitutional forms of participation, an equally important aspect is leveraging the context in which the survey took place. Societies rarely experience such a radical shift in the issue agenda as the one brought by the Covid-19 pandemic, allowing me to observe how existing participation patterns are altered under the influence of a mostly exogenous stimulus. Taking up noninstitutional participation during the first phase of the crisis (2020–2021) in the case of Germany, the paper benefits from testing the proposed framework in a context in which the issues of participation transform. The new, crisis-related issues are less embedded in the agenda of existing mobilizing actors or partisan identities (e.g., Lehmann & Zehnter, 2022). Therefore, differences in citizens’ participation can capture an issue effect that is less contingent on habitual participation behavior.

As the results show, during the crisis period, the protest arena played a central role in political mobilization. The paper zooms in on the variation between individual participants with various profiles and argues that an insider-outsider divide characterizes participation in non-institutional forms. Issues that have long been mobilized attract “political insiders” who are embedded in the organizational landscape of the dominant cleavage dimension. In contrast, participants on newly mobilized Covid-19 related issues resemble the image of “political outsiders” who exist at the periphery of this organizational landscape. In this regard, the issue of engagement affects the level of participation and explains differences in the profile of participants.

## Theoretical Framework

### Political Insiders and Outsiders

The study of noninstitutional participation draws on two research traditions. The first uses general-population surveys and emphasizes conventionalization as a key component of normalization. This research tradition shows that those who participate in noninstitutional forms are on average younger, more likely to be male, better educated, more politically interested, somewhat less trusting towards the state than voters, and more ideologically left-wing (e.g., Borbáth & Gessler, 2020; Dalton et al., 2010; Hooghe & Marien, 2013; Lahusen & Bleckmann, 2015; Norris et al., 2005; Saunders, 2014). The conventionalization literature suggests that some of these differences—particularly the socio-demographic ones—shrink or even disappear over time, as protest becomes normalized and part of the action repertoire of a broad segment of society. As far back as 20 years ago, Aelst and Walgrave (2001) showed that demonstrations had become a form of political participation that is increasingly available to a wide variety of groups. In this context, they introduced the idea of the normalization of protesters and observe that with the partial exception of less affluent people with low education, all social groups were increasingly present in public demonstrations. Looking at Germany, Lahusen and Bleckmann (2015) compared the predictors of participation in lawful demonstrations in 1974/75 using the Barnes and Kaase (1979) dataset with the European Values Study-EVS (1981, 1990, 1999, 2008). In line with the normalization of the protesters hypothesis, they found that educational and class-based inequalities in participation considerably shrunk over time.

The second strand of literature uses on-site surveys and emphasizes differentiation as a key component of normalization. Notwithstanding a trend of conventionalization, the differentiation literature documents how features of the meso-level mobilization context, typically of demonstrations, are associated with enduring attitudinal differences both between (e.g., Daphi et al., 2021; Grasso & Giugni, 2019) and within “crowds” (e.g., (Saunders, 2014; Saunders & Shlomo, 2021). The largest effort to comparatively survey demonstration participants has been the “Caught in the act of protest: Contextualizing contestation” research project (CCC, van Stekelenburg et al., 2012). CCC analyzed participation in 71 leftwing demonstrations in Spain, Italy, the Netherlands, Belgium, Sweden, Switzerland, and the UK. Focusing on the distinction between old and new left events, Giugni and Grasso (2019) show that those who demonstrated about old-left (economic) “bread and butter” issues were more likely to come from a working-class background than those who demonstrated about new-left (cultural) issues. The latter group was more likely to be from a more privileged socio-economic background, had a different set of values (Grasso & Giugni, 2019) and was closer to party politics than their old-left counterparts. Exploring the variation within participants in the CCC data, Saunders (2014) showed that participants can be distinguished based on their frequency of participation in demonstrations according to the extent to which they trust and participate in formal institutions and electoral politics. The results demonstrated that—with the partial exception of “stalwarts”, the group that most regularly participated in demonstrations—all others were likely to combine noninstitutional and electoral engagement. In the context of Germany, Daphi et al. (2021) showed variation between crowds of participants in nine different demonstrations, organized from 2003 to 2020. They found two clusters of participants, distinguished by governmental trust, satisfaction with democracy, efficacy, perception of nonrepresented, and nonresponsiveness. Although they do not interpreted their results along a political insider and outsider divide, these studies showed that politically under-represented groups also participate in noninstitutional forms.

In examining the extent to which there is an emerging insider and outsider divide much depends on how the terms are defined. There are two dominant conceptualizations in the scholarly literature.^1^ On the one hand, the political economy tradition uses the insider-outsider distinction to differentiate those in secure employment from the ones who are unemployed, or employed in atypical or precarious jobs (Schwander & Häusermann, 2013). On the other hand, without using the exact terms, the political mobilization literature relies on a similar dichotomous distinction between dominant and challenger parties, depending on whether they have previously been in government or not (de Vries & Hobolt, 2020). Both traditions have in common the focus on the positionality of actors vis-à-vis broader power-dynamics. In the case of individuals’ labor market position, the distinction is used to examine the link to established institutions, be that trade unions or political parties, that shield insiders by representing the interests of the group they belong to. In the case of parties, the distinction is used to map the programmatic strategies newcomers employ to innovate and challenge the center (de Vries & Hobolt, 2020, pp. 53–58). Newcomers become dominant actors by ‘issue-entrepreneurship’, introducing new lines of conflict, and transforming existing cleavage structures (also see: Kriesi et al., 2012).

Taking up the idea that actors’ positionality vis-à-vis established institutions, and broader societal lines of conflict is a key dimension in protest politics as well, I propose to distinguish insiders and outsiders on a continuum, according to the extent to which they are embedded in the organizational landscape of the dominant cleavage dimension. “Embedded in” refers to an attitudinal and a behavioral element. In terms of the attitudinal element, I define political insiders as individuals who trust state institutions, and take a moderate position on the primary ideological dimension, away from the extremes. In terms of the behavioral element, I define political insiders as individuals who are being represented by dominant parties, that have previously been in government. I also consider being a member in a dominant civil society organization a component of the behavioral element of being a political insider. Political outsiders are defined in opposition to insiders; they are a group that is typically associated with “exit” instead of “voice” (e.g., Verba et al., 1995).^2^

Building on the political mobilization literature, I examine the potential of outsiders to challenge the status quo. In the case of political parties, as key agents of conflict mobilization, their outsider potential is manifested in challenger mobilization. In contrast, individuals, who resemble outsiders, only challenge the system when they are politically mobilized. It is an open empirical question whether outsiders participate as challengers or become politically alienated, with an unmobilized ‘challenger potential’.

Following the perspective of differentiation in protest politics, I expect that political outsiders rely on noninstitutional forms to voice their grievances on issues important to them. This challenges the dominant view of normalization as conventionalization, and emphasizes the diversity of protest participants, where next to insiders, outsiders also participate. Accordingly, I formulate the first hypothesis as:

#### Hypothesis 1

Political outsiders participate in forms of noninstitutional participation to a similar extent as political insiders.

### The Covid-19 Crisis and Issue-Specific Engagement as the Missing Link

The dominant perspective of activity-, instead of actor-centered participation research (Oser, 2017, 2021), shifts the focus away from variation in the profile of protest participants. Re-focusing on the issue on which individuals participate enables formulating expectations on differentiated participation by political insiders and outsiders. In this regard, the current section introduces and applies the distinction between established and new issues to Germany’s protest arena. While the set of issues that emerge as established or new is specific to this context, the underlying logic travels to other arenas of participation (e.g., electoral or institutional forms), as well as to alternative geographical, or temporal contexts. Issues of mobilization provide the missing link between micro and meso level features, and give an opportunity to integrate some of the insights of the literature on protest event analysis in individual participation research (e.g., Fisher et al., 2019; Hutter, 2014; Hutter & Borbáth, 2019; Kriesi et al., 2012).

From a long-term perspective, protest in Western Europe went through a transformation when new social movements rose in the second half of the 1970s and has since been the home arena of progressive mobilization on cultural issues (Hutter, 2014; Hutter & Borbáth, 2019). Germany is a case that is emblematic of the transformation, with the overwhelming majority of protest events being organized on so-called “cultural” issues (Hutter, 2014, p. 102). In particular, mobilization on two issues stands out. The first of these is climate protection, which goes back to the anti-nuclear movement in Western Germany (Rucht, 1990), and the second is anti-racism/ immigration, the main issue in protest after re-unification (Hutter, 2014, p. 107). These two issues—climate protection and anti-racism—are established issues, with continuing mobilization during the pandemic by contemporary movements such as Fridays for Future, or Black Lives Matter.

Notwithstanding this long-term perspective, the short-term dynamics of the Covid-19 crisis represent an apparent rupture. On the one hand, due to the institutional measures introduced to fight the pandemic and new social norms around contact restrictions, demonstrations in public spaces became difficult and not always allowed. In this regard, the crisis is expected to halt political participation. On the other hand, new and unprecedented rules regulating working from home, traveling, shopping, and many other areas of daily life were adopted to curb the rate of infections. The adoption of these rules led to a democratic dilemma (Engler et al., 2021) that created new types of grievances and allowed political entrepreneurs to mobilize in noninstitutional forms. From this perspective, rather than representing a halt in political participation, the crisis may have led to further conflict and mobilization.

The pandemic radically altered the issue agenda by bringing the twin issues of public health and economic assistance to the fore (Borbáth et al., 2021). There was a gradual shift in health-related engagement during the crisis, from an expression of solidarity with the medical community to engagement by those opposing the restrictions on civil liberties introduced to curb the infection rate. In Germany, the so-called Querdenker demos regularly made headlines with their opposition to restrictions on freedom and denial of the threat of the pandemic. Engagement related to economic assistance, resulting from the shutdown of the economy in the wake of the crisis, range from demanding direct financial support from the state to demands for a loosening of the lockdown rules to allow firms to re-open.

The two sets of issues—restrictions on freedom and economic assistance—are not equally new in the field of protest politics. As far as protection against the economic fallout is concerned, this represents a continuation of existing struggles against the separation of state and market. Similar connections are less clear-cut in the case of protest against the freedom restrictions mobilizing the grievances that the new rules of restrictions created. Freedom restriction protests are largely unprecedented, and mostly explained by the emergency rules adopted to curb the Covid-19 infection rates. Existing findings on participation in these protests show that participants are increasingly on the right and are differentiated by their strong belief in various conspiracy theories. At the same time, they often do not vote for radical right parties like the AfD or even do not vote at all (Grande et al., 2021). This points towards the importance of issues in explaining differential participation by political insiders/ outsiders. Accordingly, my second hypothesis concerns continuity of insider participation on established issues, and mobilized outsider participation on the new, Covid-19 related issues:

#### Hypothesis 2

Political insiders are more likely to participate in forms of noninstitutional participation on established issues such as anti-racism and environmental protection, whereas political outsiders are more likely to participate on new issues such as protests against the restrictions on freedom.

As a mechanism explaining differential participation, I highlight the role of issue preferences. In their comprehensive model of protest participation, Rucht and Neidhardt (2002) identify the formation of “movement milieus” brought about by a trend of individualization and modernization as the main driving force behind differentiation in protest politics. At the micro level, their model identifies issue preferences that stand for politically interpreted grievances. However, micro-level preferences and grievances are affected by context level features (Grasso & Giugni, 2016) and potentially change over time. By not linking participation to its meso-level context of mobilization, existing studies generally miss the dynamic of within-individual change over time, and instead assume that the individual-level propensity to protest stays constant (with some exceptions, e.g.,: Finkel & Muller, 1998).

Yet, if we wish to disentangle the mechanism behind issue-specific participation, change over time may be important. Participation is potentially driven by both the changing dynamic of issue-specific preferences/ discontent and by the fact that certain issues are particularly mobilizing because they resonate with the identity of a specific milieu (Rucht & Neidhardt, 2002). The normative and political implications of the difference are considerable: if noninstitutional participation is also rooted in issue preferences, in addition to group identities, protest can potentially act as a retrospective accountability mechanism that develops in reaction to governmental decisions (Altiparmakis & Lorenzini, 2018; Grasso & Giugni, 2016).

The latter interpretation is consistent with an instrumental, rational choice model, where one of the central components of the individual level calculus of participation is grievances related to the provision of collective goods. However, grievances are assumed to not carry much explanatory power due to their relative over time stability (Jenkins, 1983, p. 530). Part of the reason why grievances appear constant might be explained by their operationalization, that often relies on encompassing indicators, such as satisfaction with the government, democracy, or a scale of multiple policy preferences (e.g., Bäck et al., 2011; Finkel & Muller, 1998). Compared to earlier studies, the Covid-19 crisis, during which a combination of lockdowns and easing of restrictions followed each other in quick succession and unprecedented policies were implemented, provides the ideal context to leverage the dynamic of volatile preferences on specific issues and examine the extent to which they result in varying levels of noninstitutional participation. Contrary to discourses that often exclusively associate protest participation during the Covid-19 crisis with citizens being embedded in a specific milieu, I hypothesize that:

#### Hypothesis 3

Issue preferences explain noninstitutional participation by the same individual over time.

## Methodological Approaches and Data Sources

### Methodological Challenges in Studying Noninstitutional Participation

In the previous section, I reviewed the main substantive results of studying non-institutional participation with general-population surveys and social movement mobilization with on-site surveys. The current section systematically compares them from a methodological perspective.

In their *empirical scope*, the two are complementary: General-population surveys are best fit to study the distinction between participants and nonparticipants, whereas on-site surveys are designed to capture differences between participants. In general-population surveys, those who participate in noninstitutional forms are “rare cases”. For instance, only three European countries report shares of people who demonstrated in the last 12 months of above 10% (Italy, France, and Spain; on average across the 1–9 European Social Survey-ESS waves). With a typical sample size of around 1000, this leaves a sample of 100 demonstrators, a number too small to reliably capture differences among participants. As a partial solution, researchers often construct a scale of noninstitutional participation and lump together participation across different forms (e.g., Hooghe & Marien, 2013). However, forms that vary in contentiousness might have different predictors that potentially undermine the estimation strategy. On-site surveys solve this by sampling only those who participate in demonstrations. While such samples reflect the diversity of demonstrating crowds, they are not fit to compare between participants with nonparticipants and between participants in different forms.

The two approaches differ in their sampling frame: general-population surveys aim to represent the socio-demographic characteristics of the society from which respondents are sampled, while on-site surveys aim to represent participants of the demonstration during which respondents are sampled. The demonstrations studied are not randomly selected: They are larger events in bigger cities that are easier to access for research purposes. In addition, on-site surveys face the problem of differential nonresponse-participants in right-wing rallies are less likely to answer the survey (Daphi et al., 2015). Given these biases, on-site surveys are limited in their capacity to represent the dynamic in the protest arena as a whole.

The two approaches also differ in how data is collected and the *context of the interview*. Whereas in on-site surveys, respondents are interviewed during a political act (demonstration), interviews in general-population surveys are undertaken outside of such a context. As Saunders (2014, pp. 586–587) argues, the different contextuality might result in over-reporting of some of the political variables in on-site surveys, for instance, correlates of political interest.

In general-population surveys, the *time horizon* of individual survey items plays a crucial role. These surveys offer no direct information on the mobilization context, the temporal anchor in the survey item is the only way to match individual behavior to its context of mobilization. However, the main comparative general-population surveys make different choices in this regard. While some include a time reference—they ask respondents whether they have engaged in various noninstitutional participation forms—others include no time reference and thus render it impossible to include contextual factors based on the time of the reported behavior. These are highly influential choices, since as Robison et al. (2018) demonstrates, large comparative surveys set the agenda of participation research for decades. On-site surveys only face a similar issue when they ask respondents to recall how often they had participated in previous demonstrations. While similar recall items are to some extent biased, they allow researchers to compare first-time, regular, and occasional participants (e.g., Giugni & Grasso, 2019; Saunders, 2014; Verhulst & Walgrave, 2009), and zoom in on an empirical dimension that is difficult to capture with general-population surveys.

In general-population surveys, one can only indirectly infer what networks respondents are most likely embedded in and what type of issues they are likely to get engaged in based on individual-level features. In this regard, left-right self-placement and organizational membership might serve as valid proxies. In contrast, on-site surveys have the critical advantage of directly observing features such as the issue or the mobilization network, even if they have difficulty surveying right-wing events. As the discussion shows, the two approaches make different methodological choices with distinct advantages and disadvantages. To summarize, Table 1 lists how the two differ along the dimensions discussed above.

**Table 1:** Two methodological approaches in the study of political participation

	General-population surveys	On-site surveys
Empirical scope	Distinction between participants and nonparticipants	Distinction between participants
Unit of analysis	Survey respondents, nested in a country/year context	Survey respondents, nested in an event in a country/year context
Sampling frame	General population	Participants in a particular event
Context of the interview	Devoid of context	Context of a political act
Time-horizon	Specified (e.g., last 12 months) or unspecified (throughout the life course)	Recall of retrospective participation (in previous events)
Issue of mobilization	Typically indirectly inferred: e.g., left-right self-placement of the respondent	Directly observed from meso and event-level features
Network of mobilization	Typically indirectly inferred: e.g., organizational membership of respondents	Directly observed from meso and event-level features
Form of participation	Extensive lists of various forms	Public demonstrations

### Combining the Best of Both Worlds: A Novel Survey Item

In the current section, I introduce my methodological approach and the survey items I rely on. The questionnaire aims to bring the advantages of onsite surveys to a representative general-population survey to bridge the two methodological traditions of political participation research. The survey has been designed and implemented in the context of the research project Civil Society Potentials: Solidarity in Crisis Management (SolZiv),^3^ funded by the Berlin University Alliance.

The survey was conducted among members in an online access panel in Germany between October 14 and November 4, 2020 with respondents recruited by the survey company *Respondi*. The data was collected with quotas for age, gender, education, and region (east-west), based on official statistics from Eurostat for 2020 and is representative of 18- to 69-year-old German residents.^4^ We aimed to collect a large sample to be able to study differences both between nonparticipants and participants, as well as among participants. In total, the sample includes 3330 respondents. Four months later, between March 2 and March 11, 2021, 1004 respondents were re-interviewed in a panel design.^5^ We applied the same quotas to ensure that the re-interviewed respondents were representative in terms of age, gender, education, and region. In addition, I rely on nonresponse weights to ensure that the re-interviewed sub-sample did not significantly differ from the original sample. Online Appendix D includes further information on the weighing procedure.

Early on in the survey, respondents were asked to recall the time frame between the first lockdown in March 2020 and the survey interview.^6^ Then, they were asked to indicate their frequency of engagement using a five-point scale: (1) Never; (2) Rarely; (3) Sometimes; (4) Often; (5) Very often. Online Appendix C presents the exact formulation of all survey items, but the listed forms of political engagement were: (1) public protest activity; (2) illegal public protest activity; (3) online protest activity (i.e., digital protest); (4) posting about politics online; (5) signing a petition; (6) contacting a politician; (7) activities of political parties; or (8) other forms.

**Fig. 1:**
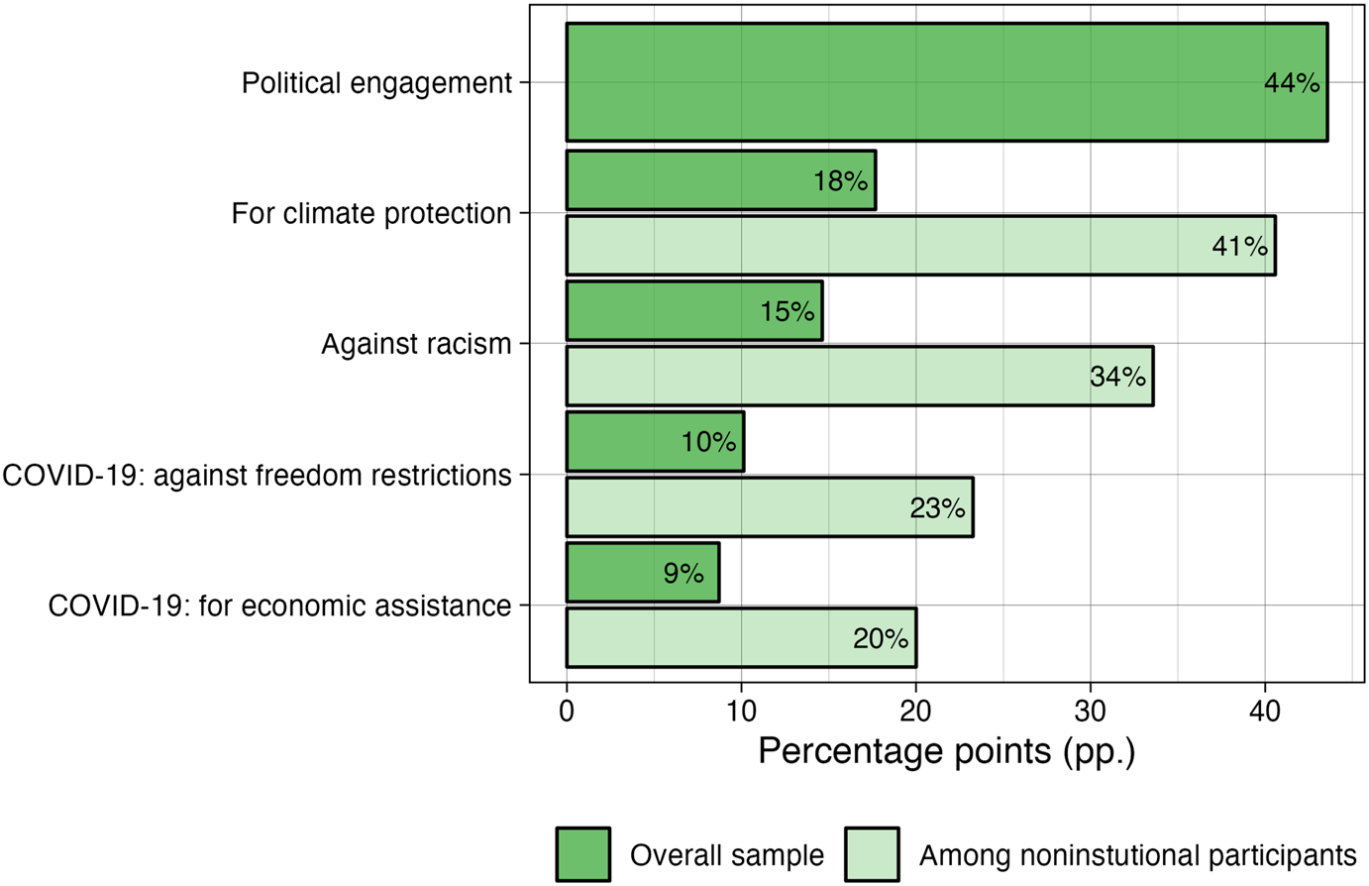
Issues of noninstitutional participation. ﻿Issues of engagement are not mutually exclusive – a respondent could indicate participation on more than one issue. Results are based on the first wave, weighted by the socio-demographic weight

I follow the definition of noninstitutional participation as set out by (Hooghe & Marien, 2013, p. 139, also see Barnes & Kaase, 1979), according to which these forms are *“used predominantly by nonelite actors, in order to challenge the political elite or to gain access to the political agenda”*. In contrast, institutional forms are *“defined and organized by members of the political elite (most notably political parties)”*. Although, institutional and noninstitutional forms are often combined (e.g., Jeroense & Spierings, 2022; Oser, 2017, 2021), numerous empirical analysis (among others by Hooghe & Marien, 2013) demonstrates that they are associated with different predictors and as van Deth (2014) argues, represent theoretically distinct modes of participation. Following the Hooghe and Marien (2013) operationalization, I rely on the first five forms as indicators and exclude “contacting a politician”, “party activities”, and “other forms”. Figure 1 in Online Appendix A shows the level of participation in noninstitutional forms.

In a second follow-up question, respondents were asked to indicate the issue on which they got politically engaged. The question was posed to all respondents who answered that they took part in any of the above forms. The respondents could select issues on which they got engaged from a closed list of the ones that dominated protest during this period: (1) against racism; (2) for climate protection; (3) against limitations to freedom due to the coronavirus crisis; (4) for governmental economic help due to the coronavirus crisis; (5) other issues.^7^ The item allowed selecting multiple issues. The handful of issues do not aim to be representative of all issues that potentially motivate noninstitutional participation. They were selected to represent the most prominent issues on which the highest number of participants were engaged in protest in Germany right before and during the Covid-19 crisis. Figure 1 shows its distribution both in the overall sample and among participants in noninstitutional forms.

In line with the normalization perspective, engagement levels between March and October 2020 were relatively high: 44% of German society was politically engaged in some form. The most prevalent is engagement for climate protection, followed by engagement against racism. The share of the latter reflected recurring engagement on immigration as well as on the Black Lives Matter protests. Beyond these two established issues, there was relatively high engagement on the two new issues related to the Covid-19 pandemic. Roughly a fifth of those who participated were engaged in protesting against restrictions on civil liberties or for economic assistance.

From this data structure I constructed three dependent variables that I relied on in the different types of analysis. Firstly, to test H_1_, I focused on the driving forces of participation. I took up the idea from the literature using on-site surveys on structural differences between participants based on the frequency of their participation and distinguished between overall participants and regular participants. Overall participation was a dichotomous measure, distinguishing those who participate in at least one of the five noninstitutional forms, whereas regular participation distinguished those who “often” or “very often” participated in any of these five forms. Secondly, to test H_2_, I moved away from the forms of participation and relied on latent class analysis of the different issues of participation (for recent applications of LCA in political participation research in Europe see: Jeroense & Spierings (2022), and Oser (2021) in the US). Following a one-step approach, I estimated a latent class model with covariates (Bolck et al., 2004). Thirdly, to test H_3_, I utilized the second wave of the data collection and conducted a series of logistic regression models with individual fixed effects, where the dependent variable is dichotomous issue-specific participation, estimated for noninstitutional participation for climate protection, against racism, against limitations on freedom, and for governmental economic help.

The key independent variables referred to the operationalization of the insider-outsider status, distinguished according to the extent to which they are embedded in the organizational landscape of the dominant cleavage dimension. For the attitudinal dimension, I rely on two indicators: 1. trust in state institutions (5 points), and 2. extremism of the left-right position. I rely on left-right as a heuristic, super-issue that summarizes positions on multiple dimensions (Inglehart & Klingemann, 1976).^8^ The indicator for extremism is the squared term of the mean-centered left-right scale.^9^ For the behavioral dimension, I also rely on two indicators: 1. vote choice (CDU/CSU as the reference category); 2. membership of civil society organizations (9 points). Based on the vote choice question, in which respondents were asked to identify a party he/she would vote for if national elections were taking place next Sunday, I construct a dichotomous indicator for being represented by a dominant party. The indicator takes the value of one for parties that have previously been part of the federal government, such as CDU/CSU, SPD, B90/Grüne, FDP; and a value of zero for challenger parties, such as the AfD, die Linke, another party, or nonvoting.^10^ For membership of dominant civil society organizations, I calculated the number of organizations the respondents indicates that they have been a member of, from a list of nine organizations, excluding “patriotic alliances” and “other types of organizations” (also see Online Appendix C).^11^

A second set of independent variables refers to issue preferences. Respondents were asked to indicate how concerned they are about the protection of the environment (3 points scale) and how much they agreed with the statement *“Increasing diversity is threatening life in Germany in general”* (5 points scale). They were also asked two positional questions to indicate to what extent the policies implemented (1) to overcome the health risks of the Covid-19 crisis and (2) to overcome the economic consequences of the Covid-19 crisis were insufficient or too extreme (5 points scale).^12^ As control variables I included factors that the literature identified as potentially important in distinguishing protesters, such as political interest (4 points), left-right position (11 points), and socio-demographic characteristics. The latter includes age, age squared, having gone through higher education (2 points), subjective income (4 points), the type of residence (reference category: big cities) Depending on the model, I also controlled for the forms of participation.

Since the general-population survey the analysis relies on has both the form as well as the issue of participation, with a large-N, it allowed me to overcome some of the methodological challenges identified in the previous section. It combines the advantages of on-site surveys with those of general-population surveys by: (1) including information on the issues on which respondents are mobilized; (2) including a specified time horizon during which engagement takes place; (3) providing a scale of the frequency of participation. At the same time, some of the drawbacks of on-site surveys are avoided: (1) the results are generalizable to the protest arena during this period; (2) the interview takes place in a nonpoliticized context; (3) the forms of participation are allowed to vary beyond public demonstrations.

## Empirical Results

### Participation by Political Insiders and Outsiders

**Fig. 2:**
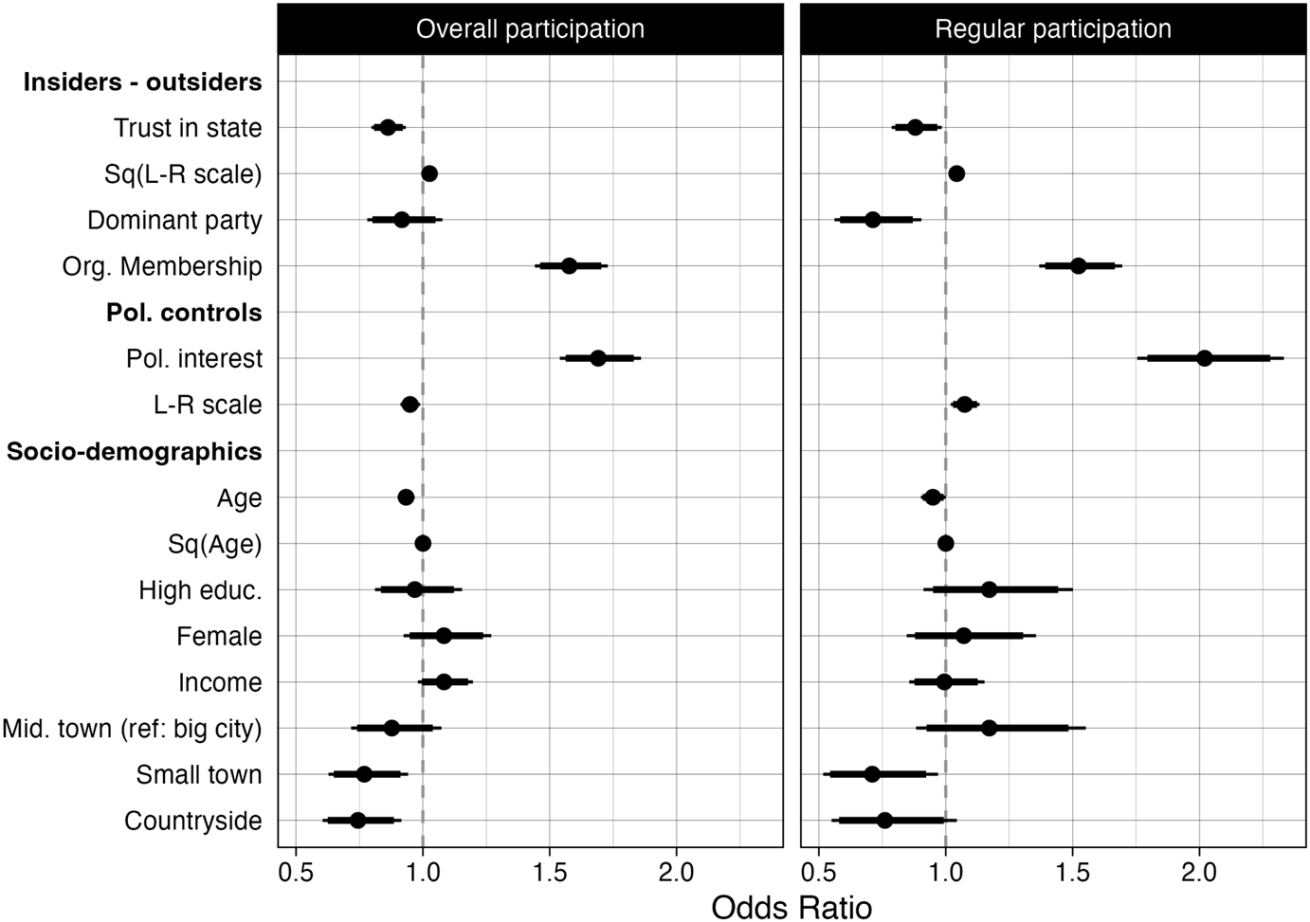
Participation in noninstitutional forms during the Covid-19 crisis in Germany. Dependent variables: overall participation & regular (often/ very often) participation. See the corresponding regression model included in Online Appendix A, Table 1. The thicker error bars represent 90% confidence intervals, the thinner error bars represent 95% confidence intervals. Results are based on the first wave, weighted by the socio-demographic weight

To test the extent to which political insiders and outsiders participate (H_1_), I construct two logistic regression models where overall and regular participation are the dependent variables. The key independent variables are the indicators of insider/ outsider status. The model also controls for political interest and the socio-demographic factors previously listed. Figure 2 shows the results, with coefficients presented as odds ratios.

As the results show, the four different indicators of political insider and outsider status vary in their effect on noninstitutional participation. Those who trust the state less and identify with more extreme left-right positions appear to be more likely to participate, indicating a presence of political outsiders in noninstitutional participation. At the same time, the effect of organizational membership points towards the prevalence of political insiders in noninstitutional forms: the probability to participate significantly increases the more organizations the respondent belongs to. Being represented by a dominant party has no identifiable effect on overall participation, voters of these parties as well as nonvoters or voters of challenger parties are equally likely to participate. At the same time, this is the main difference between overall and regular participants: among regular participants there is a higher share of outsiders, in terms of nonvoters or voters of all challenger parties (see Online Appendix B, table & figure 7). In line with the conventionalization perspective, socio-demographic factors play little to no role in explaining participation in noninstitutional forms or in differentiating regular participants.^13^

Based on this model, the predicted probability to participate for a political insider who trusts the state (90th percentile), takes a moderate ideological position (10th percentile), is represented by a dominant party, and is a member in two civil organizations (90th percentile) is 52.8%.^14^ In contrast, the predicted probability to participate for a political outsider who does not trust the state (10th percentile), identifies with the extremes on the left-right scale (90th percentile), is not represented by a dominant party, and is not a member of a civil society organizations (10th percentile) is 49.8%. These results show that both groups participate to a similar extent in noninstitutional forms, which I take as evidence for H_1_.

### Issue Specific Engagement

**Fig. 3:**
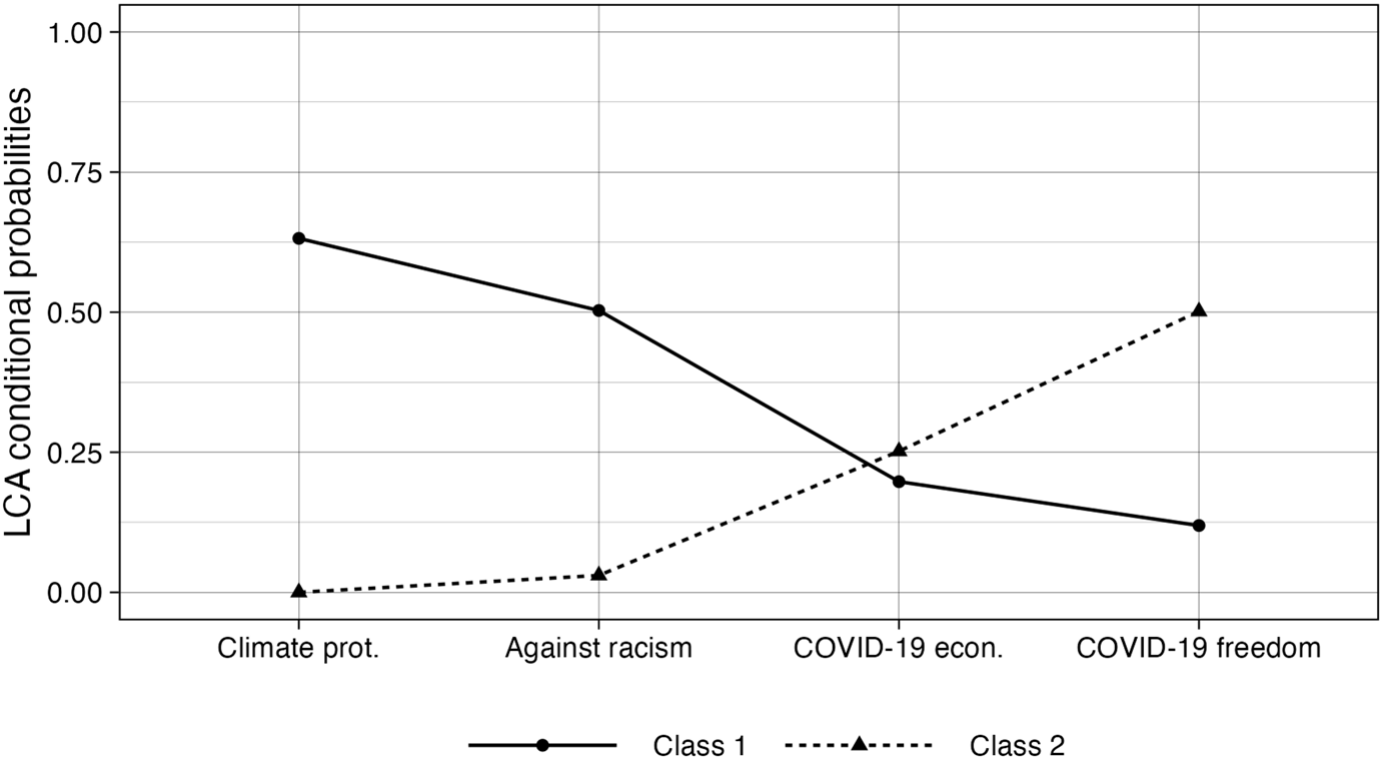
Conditional probabilities of the two-class LCA model. Issue specific engagement as a function of the class respondents belong to. See the LCA model fit information with various classes in Online Appendix A, Figure 5

As I argued in the previous sections, to the extent that political insiders and outsiders participate on different issues, issue-specific engagement provides the missing link in explaining their presence in noninstitutional forms. To examine differentiation in terms of issue-specific engagement and test H_2_, I constructed a latent class model based on the four issues that the survey distinguishes. In this analysis, I exclude nonparticipants and narrow the sample to those who have participated in noninstitutional forms. I select a model with two classes following the Bayesian Information Criterion and other indicators of model fit, as shown by Figure 5, Online Appendix A. Figure 3 shows the probability of participation on the different issues, conditional on the class the respondent belongs to.

As the figure shows, participation for climate protection clusters together with participation against racism. At the same time, participation on the Covid-19 related economic assistance clusters together with participation against restrictions on freedom. There is little overlap between the issues on which the two groups participates, with the partial exception of participation for Covid-19 related economic assistance. Those who participate on the established issues of climate protection and against racism to a limited extent also protest for economic assistance. However, to a lower extent than those who participate against the restrictions on freedom. In this regard, the results underscore the distinction between established and new issues. Based on the model predictions for the sample, 58.8% of noninstitutional participants engaged on the established issues (class 1), and 41.2% of noninstitutional participants engaged on new issues (class 2).

**Fig. 4:**
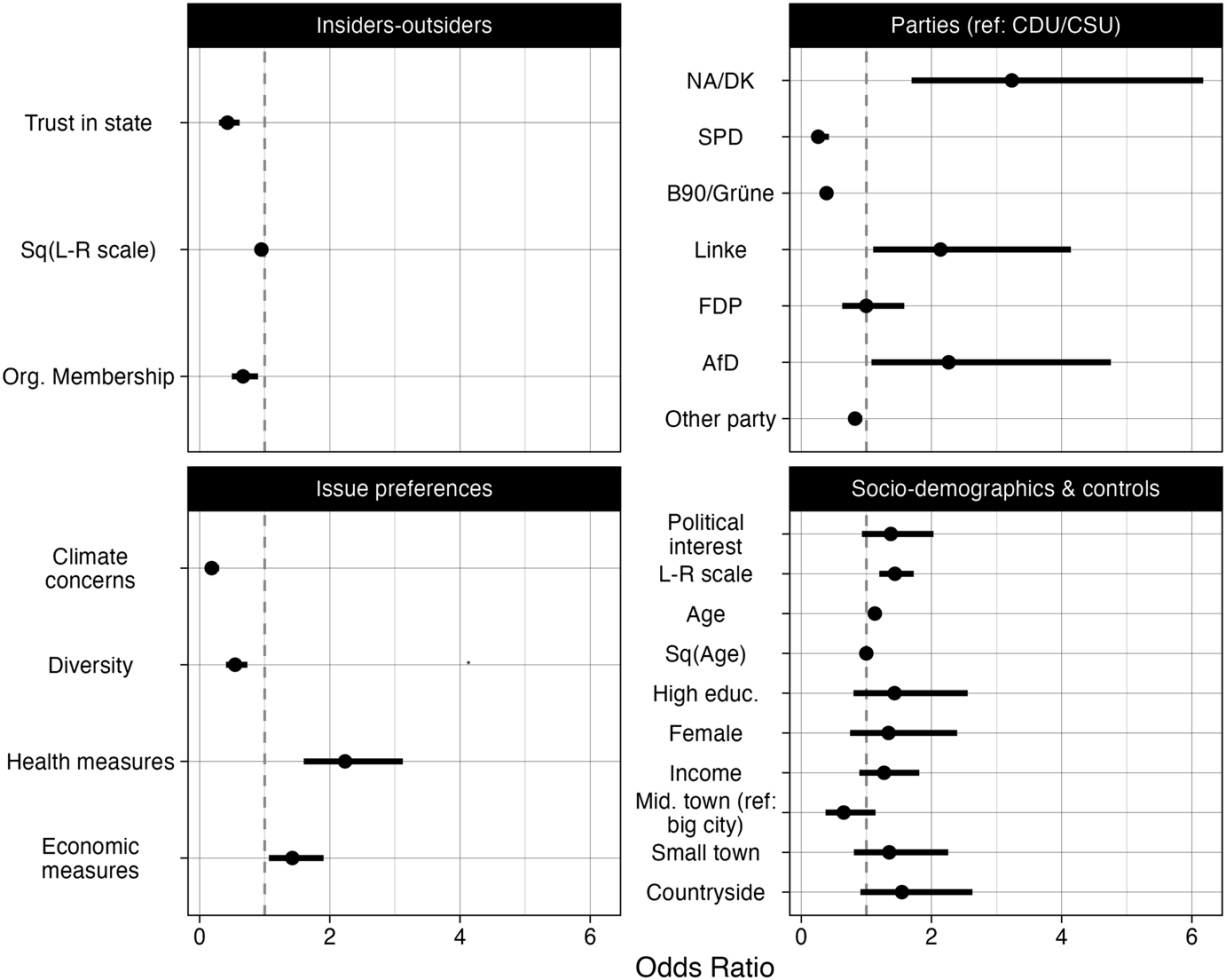
Latent class analysis of individual differences in issue-specific participation. Dependent variable: membership in the class of new issue participants (ref: established issue participants). Controls for the form of engagement. See the full regression in Online Appendix A, Table 2. The error bars represent 95% confidence intervals

In the next step, I include covariates for membership in the two different classes, and re-estimate the model. To test H_2_, I include the indicators of political insider/ outsider status, issue preferences, forms of participation, political interest and socio-demographic controls. The dependent variable is belonging to the class of new issue participants, as compared to belonging to the class of established issue participants. I ran two logistic regression models, the first one with the dichotomous measure of being represented by a dominant party, the second one with a differentiated vote choice indicator. Figure 4 shows the results.

In line with my expectations and H_2_, political insiders, with a higher degree of trust in the state, moderate left-right positions, membership in civil society organizations, and preference for dominant parties—in particular the Greens and the SPD—are more likely to participate on established issues. In contrast, political outsiders, with a lower level of trust in the state, more extreme left-right positions, lack of membership in civil society organizations, and preference for nonvoting or for voting for challengers like the AfD/ Linke are more likely to participate on new issues. These results indicate that in line with H_2_ political insiders and outsiders engage on different issues.^15^

Beyond the political insider/ outsider divide, the cross-individual variance confirms that noninstitutional participation is rooted in issue preferences. The corresponding issues preferences are significant predictors of class membership: climate concerns and diversity for participation on established issues, health measures and economic concerns for participation on the new issues. Socio-demographic differences do not differentiate between the two classes, except those who engage on the established issues of climate protection and against racism tend to be somewhat younger, than those who engage on the new Covid-19 related issues. The forms of participation are by and large balanced across the different issues (also see Figure 2, Online Appendix A) and equally present in the participation repertoire of the two classes, although those who engage on the established issues are more likely to sign petitions than the ones who engage on the new issues.

### The Dynamic of Engagement

**Fig. 5:**
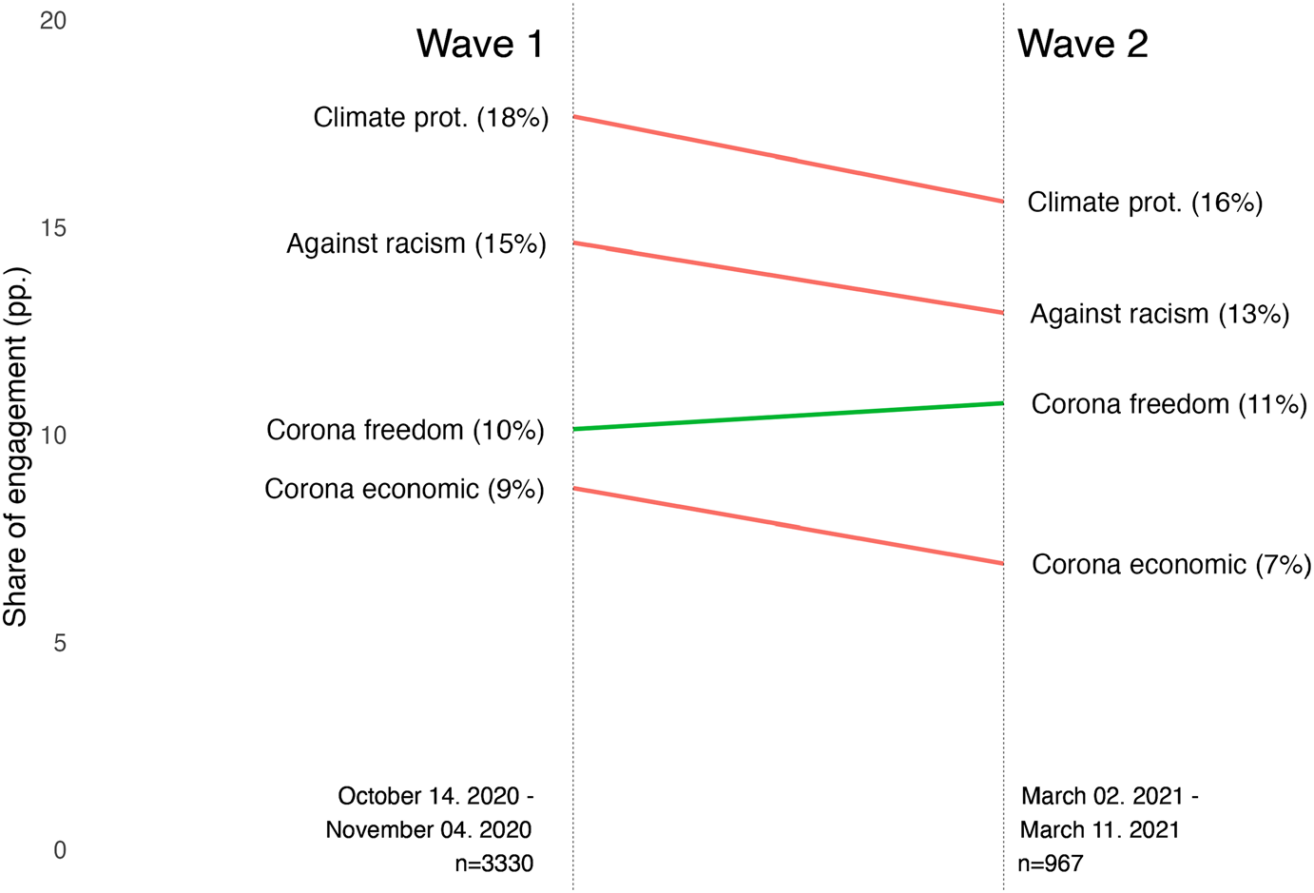
Issue-specific engagement over time in noninstitutional forms. The shares reflect the level of engagement in the overall sample. Results are based on the first and second waves, weighted by the socio-demographic and nonresponse weights

To examine the extent to which engagement is rooted in more volatile issue preferences in addition to long-standing identities, I turn to the dynamic component of the analysis. Figure 5 shows that on the aggregate level in the two waves of data collection, the level of engagement in noninstitutional forms stays remarkably stable, despite more stringent rules being introduced between November 2020 and March 2021 than they were in place during the late spring and summer in 2020.

Only against restrictions on freedom there is a slight increase in political engagement. The most significant decline is in engagement against racism, and for climate protection reflecting the shift in the protest agenda that the Covid-19 crisis brought. Nevertheless, the level of change on all issues is minimal, and overall political participation only marginally declines, from 44% in the first wave to 41% in the second wave.

**Fig. 6:**
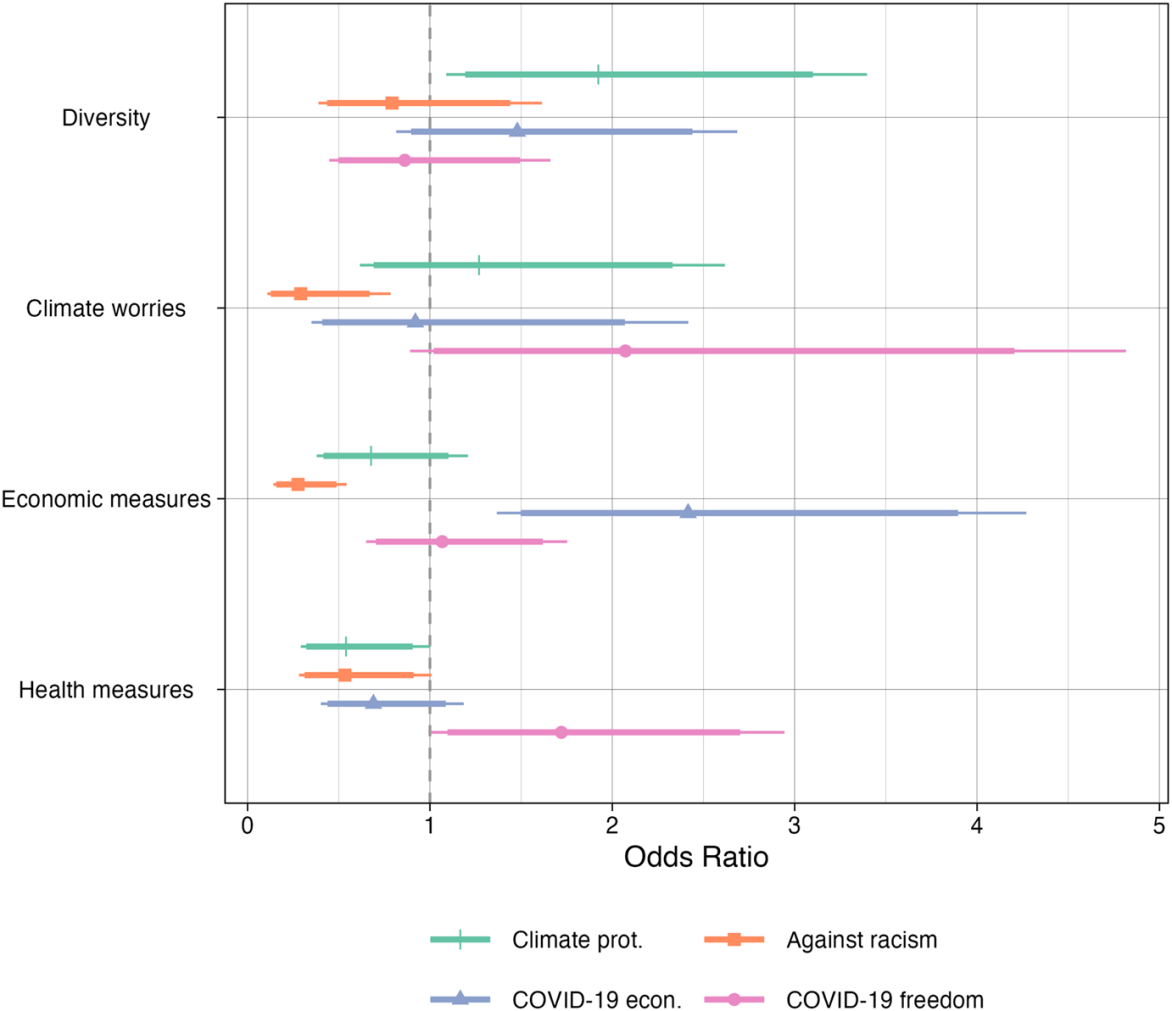
Fixed effects model of issue-specific engagement. Dependent variables: noninstitutional participation on specific issues. Controls for political interest, left-right self-placement, and trust in the state. See the corresponding regression model included in Online Appendix A, Table 3. The thicker error bars represent 90% confidence intervals, the thinner error bars represent 95% confidence intervals

However, the aggregate level of stability does not necessarily imply the lack of individual-level change. To examine changing levels of participation and test H_3_, I model issue-specific engagement with individual fixed effects. The fixed-effects model accounts for observed and unobserved cross-individual heterogeneity and allows me to isolate the role of change in issue preferences in driving engagement, net of differences in long-standing political identities and organizational embeddedness. To do so, the model only considers individuals with over time change and controls for the time changing factors of political interest, left-right self-placement, and trust in the state. The model is separately estimated for engagement on the four different issues. Figure 6 presents the results.

As the results show, issue preferences—particularly regarding the policies implemented in the context of the Covid-19 crisis—play an essential role in explaining within-individual change over time. A shift in the direction of seeing the rules to protect public health as too extreme is associated with engagement against the restrictions on freedom. A shift in the other direction, towards seeing these rules as not going far enough, is associated with increased engagement against racism. Preferences regarding economic issues shows a similar effect on engagement: those who shift towards seeing them as not going far enough are increasingly engaged for economic assistance. A shift towards seeing them as too restrictive is associated with engagement against racism. Although, preferences on diversity and the climate are more stable over time (see Online Appendix A, Figure 4), a shift towards a stronger disagreement with the idea that diversity is threatening life in Germany is associated with higher engagement with climate protection and being more worried about the climate leads to lower engagement against racism. This result highlights once more the strong inter-relationship between engagement on climate protection and against racism. I take the overall pattern as evidence for H_3_ on the role of issue preferences in driving political engagement, especially on Covid-19 specific issues.

## Conclusions

The paper bridges the noninstitutional participation research strand with the social movement studies tradition by examining individual-level participation as a function of the issue of engagement, using a representative general-population survey. It makes a substantive and a methodological innovation. Substantively, the paper introduces the conceptual distinction between political insiders and outsiders, differentiated by the extent to which they are embedded in the organizational landscape of the dominant cleavage dimension. Using the dichotomy, it shows that outsiders participate in protest politics, but on different issues than insiders. I consider the analytical distinction between political insiders and outsiders to travel beyond the protest arena, and allow to examine electoral or other forms of participation as well. Methodologically, it utilizes a new survey item to examine issue-specific engagement and to link individual-level behavior with the meso-level supply of mobilization. The context of the Covid-19 crisis allows me to map engagement on previously nonexistent issues, where habitual participation behavior linked to partisan identities and membership in networks of mobilization play a less clear-cut role.

The results show that political insiders—who trust the state institutions, take moderate ideological positions, are represented by dominant parties with government experience, and belong to civil society organizations—participate on the established issues of climate protection and anti-racism. Political outsiders—who trust the state less, take more extreme ideological positions, are nonvoters or voters of challenger parties, and are less embedded in civil society organizations—are particularly likely to engage on the new Covid-19-related issues, especially against restrictions on civil liberties. The paper demonstrates that noninstitutional engagement is rooted in the dynamic of issue preferences. In this regard, protest participation serves as an accountability mechanism in time periods between elections, and it also allows nonvoters to voice their concerns.

The perspective on the protest arena presented in this paper, namely that differentiated groups of individuals participate as a result of issue-specific mobilization, challenges a narrow understanding of normalization equated with conventionalization (e.g., Aelst & Walgrave, 2001; Lahusen & Bleckmann, 2015). At the same time, seen more generally, the Covid-19 crisis furthers the trend of normalization by providing an opportunity for political outsiders to participate in noninstitutional forms. The increasing differentiation between insiders and outsiders drives normalization to the extent that it encourages new, previously less involved participants to protest (e.g., supporters of challenger/ populist parties). However, future research should examine whether the protest arena can integrate participants with vastly different profiles and demands, or if a deep political-insider and outsider divide will emerge, resulting in “bubbles of engagement” with little interaction between participants on different issues. The empirical finding that those who protest on the new issues are often driven by grievances that are directly opposed with the grievances of those protesting established issues suggests that Covid-19-related opposition feeds into the broader dynamic of transformation of cleavage structures in Germany. In that regard, one could expect the identities formed in opposition to Covid-19 policies to structure political participation in years to come.

## Supplementary Information

Below is the link to the electronic supplementary material.

Supplementary file1 (PDF 2937 kb)

## Footnotes

1 In participation research the terms are sometimes also used to characterize action forms, as a synonym of institutional and noninstitutional participation (e.g., Jeroense & Spierings, 2022). I use them as socio-political attributes of micro-level actors, not as a synonym of their form of participation.

2 I employ these terms to capture an empirical distinction, not a normative ideal. Accordingly, I conceptualize the insider-outsider status as context-dependent and acknowledge that in some cases, groups that are from a normative standpoint ideologically radical, might become political insiders.

3 Potenziale der Zivilgesellschaft: Solidarisches Verhalten bei der Krisenbewältigung - https://solziv.blog/.

4 I also weight the analysis for these variables since not all quotas were perfectly matched during data collection.

5 In the second round of data collection we aimed to collect a random sample of about a thousand respondents, thus the reduction in the size of the sample is by design, not only due to panel attrition.

6 In the second round of data collection, both items are repeated in a slightly modified form. Instead of asking respondents to recall the period from March to October 2020, they are asked to indicate their level of engagement from November 2020 up to the interview in March 2021.

7 Other issues are excluded from the analysis, since they constitute a “class” of their own, outside of the focus of the current paper.

8 As Online Appendix B demonstrates, attitudes on the economic and on the cultural dimensions are similarly important in driving the ideological effects that the analysis uncovers.

9 Centering is applied for conceptual and empirical reasons. Conceptually, it reflects the context-dependent nature of ideological positioning, and extremism. Empirically, it is necessary to have similarly high values for extremism for those on the right (positive values), and on the left (negative values).

10 The results are also presented with a differentiated vote choice indicator, see Online Appendix B.

11 The empirical results are robust to the inclusion of these two types of organizations, they are excluded for not being dominant civil society organizations in Germany, during the respective time period.

12 I reverse the scale of climate concerns, attitude towards diversity, and the economic measures.

13 I also ran the analysis by differentiating between participants in the five noninstitutional forms, included in Online Appendix B. Although the effect sizes differ, they mostly show a similar pattern to the one presented here. Those who participate in what they themselves consider illegal protests are the most different and the least “normalized”: in their case, socio-demographic differences are also observed (older, men without higher education and a lower income), they are right-wing and trust the state more.

14 With all other control variables at their mean/ reference category.

15 As Online Appendix B shows, the hypothesis test is robust to specifying separate logit models per issue of participation instead of a latent class model.
