# Supplementary material for: Differentiation in Protest Politics: Participation by Political Insiders and Outsiders
Source: Polit Behav. 2023 Jan 12:1–24. Online ahead of print. doi: 10.1007/s11109-022-09846-7 (PMC9835032; doi:10.1007/s11109-022-09846-7)
Supplement: Supplementary file 1 — Supplementary file1 (PDF 2937 kb) [file 11109_2022_9846_MOESM1_ESM.pdf]

Appendix - Differentiation in Protest Politics Participation by Political Insiders and Outsiders

Appendix A: Additional figures and tables **1**
Descriptive figures 1
Other tables and figures referenced in the main text 5

Appendix B: Robustness checks **9**
Normalization across noninstitutional forms of participation 9
Logit replication of the LCA model 19
The effect of differentiated partisanship on participation 21
Replication with differentiated ideological scales 23

Appendix C: Survey items **27**
Dependent variables 27
Independent variables 28

Appendix D: Survey weights **33**

The replication material is available at: https://doi.org/10.7910/DVN/IBZAOJ

Appendix A: Additional figures and tables

Descriptive figures

Figure 1: Forms of participation in Germany

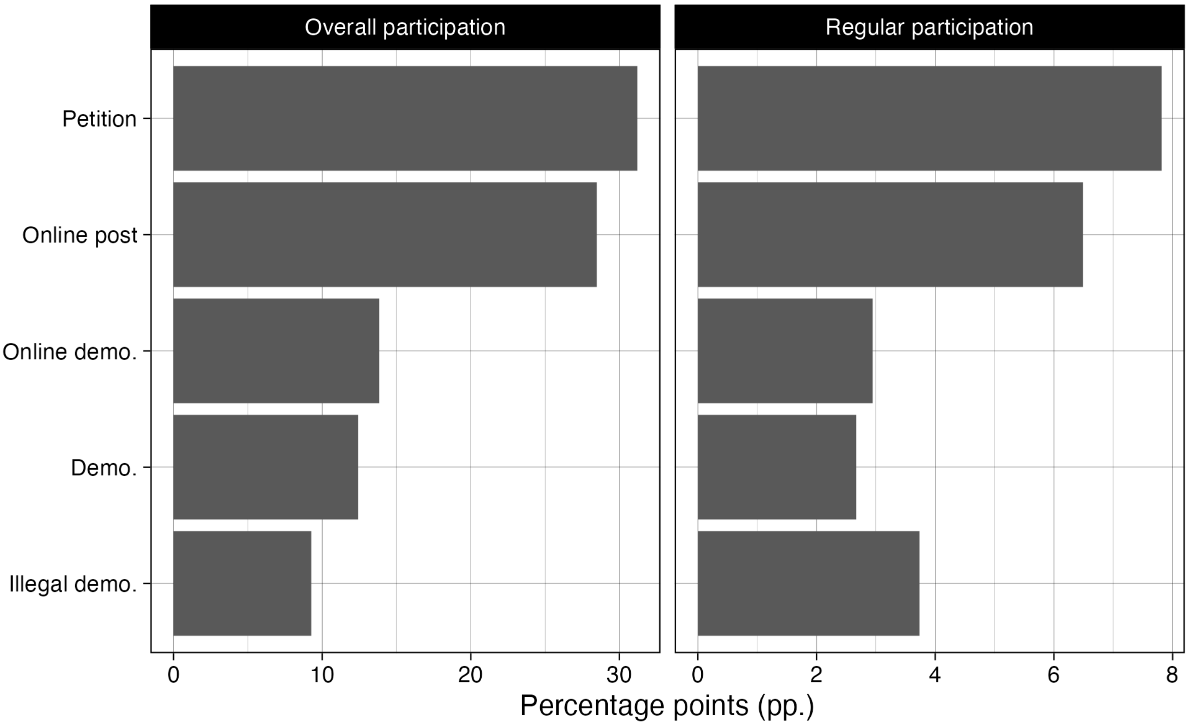

Note: Forms of engagement are not mutually exclusive – a respondent could indicate participation in more than one form. Regular engagement shows the share of those who indicate that they participated in the respective form 'often' or 'very often'. Results are based on the first wave, weighted by the socio-demographic weight.

Figure 2: Relative share of issues within different forms of participation

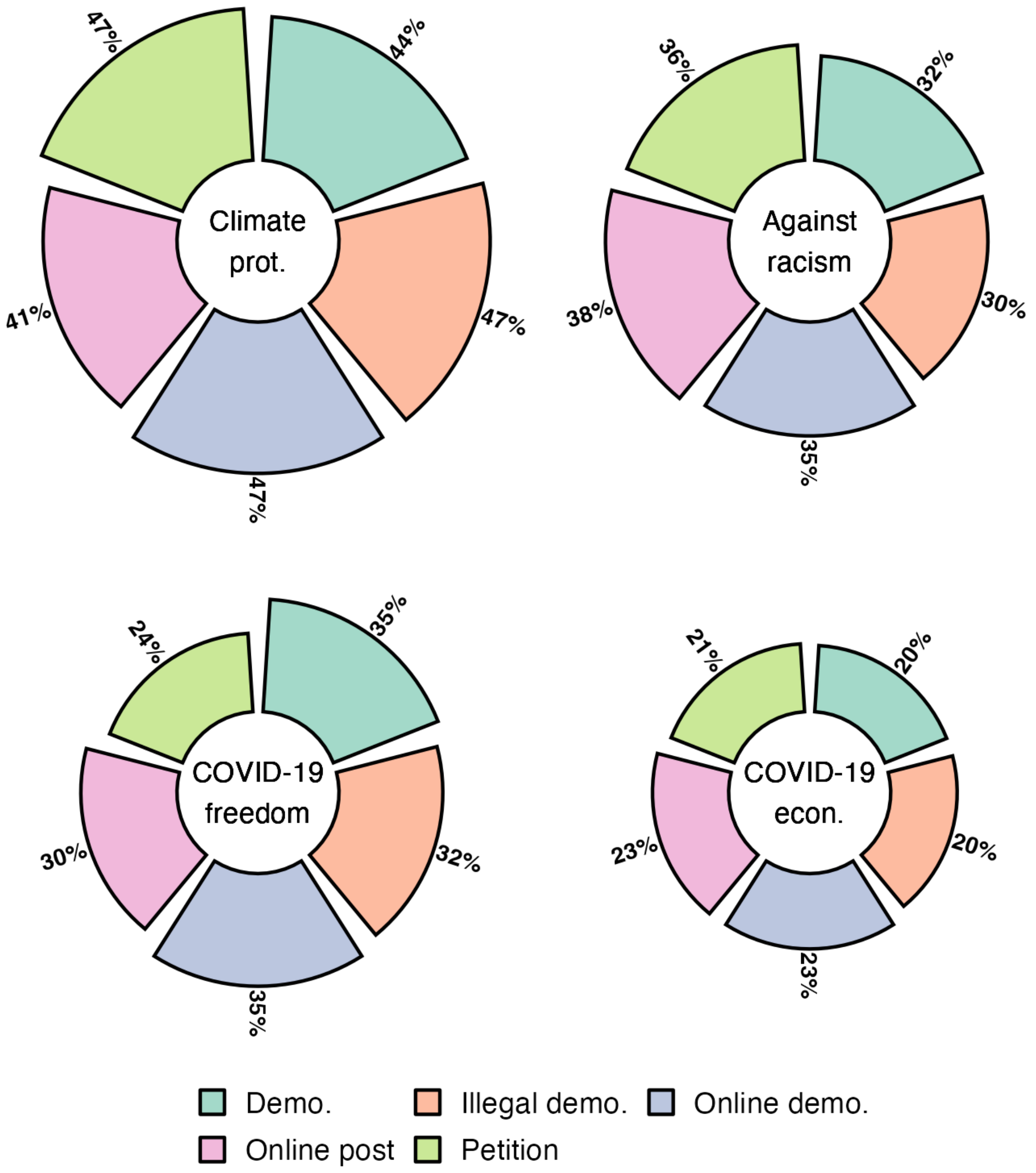

Note: The different issues do not add up to 100% within engagement forms because issues and forms of engagement are not mutually exclusive – the same respondent could indicate participation on more than one issue and in more than one form. Results are based on the first wave, weighted by the socio-demographic weight.

Figure 3: Membership in civil society organizations

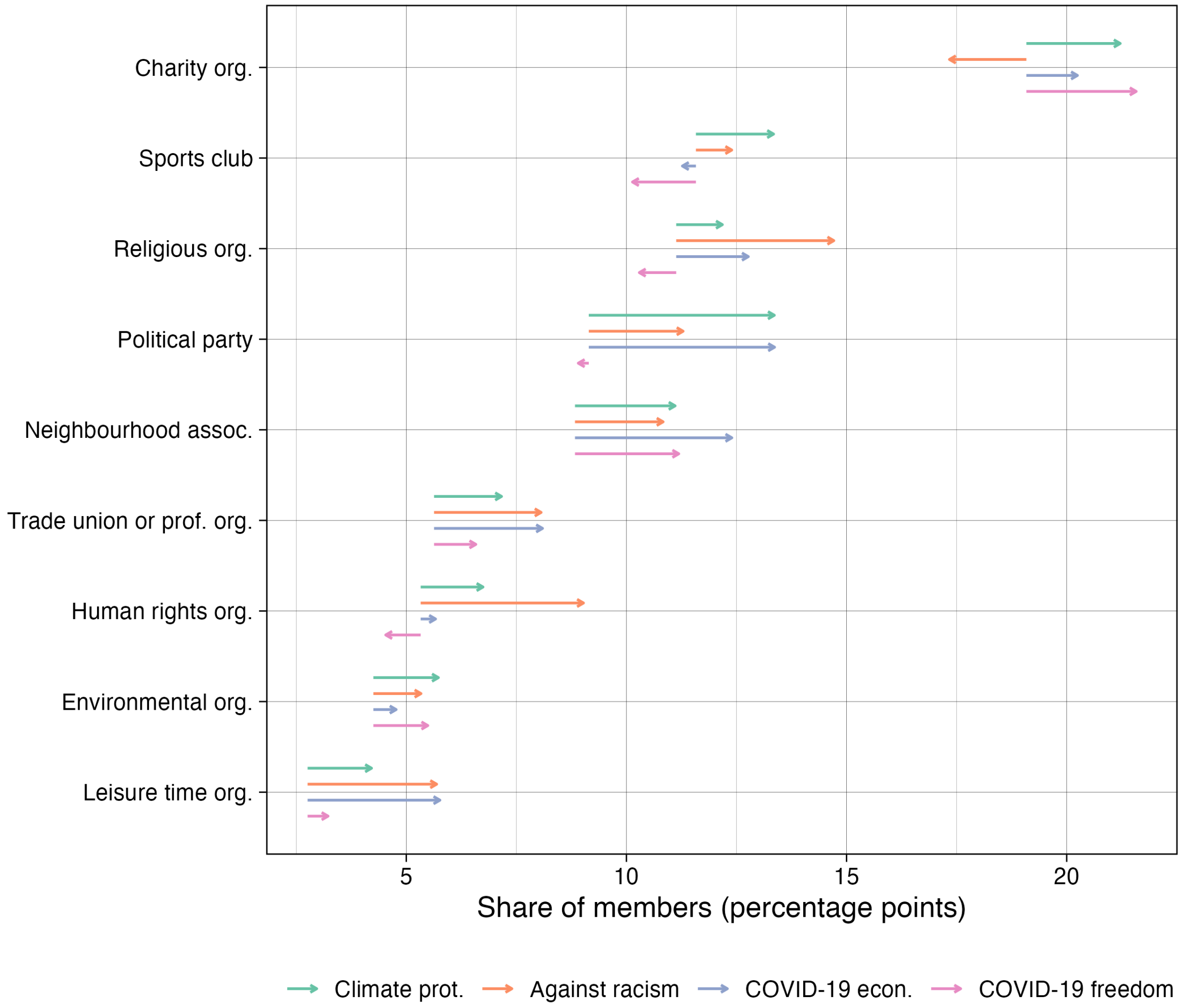

Note: The arrows show the percentage of members in civil society organizations among those who get engaged on the various issues. The reference value is the overall percentage of members among those who are politically engaged. Results are based on the first wave, weighted by the socio-demographic weight.

Figure 4: Distribution of policy evaluations in the two waves. Results are based on the first & second waves, weighted by the socio-demographic and nonresponse weights.

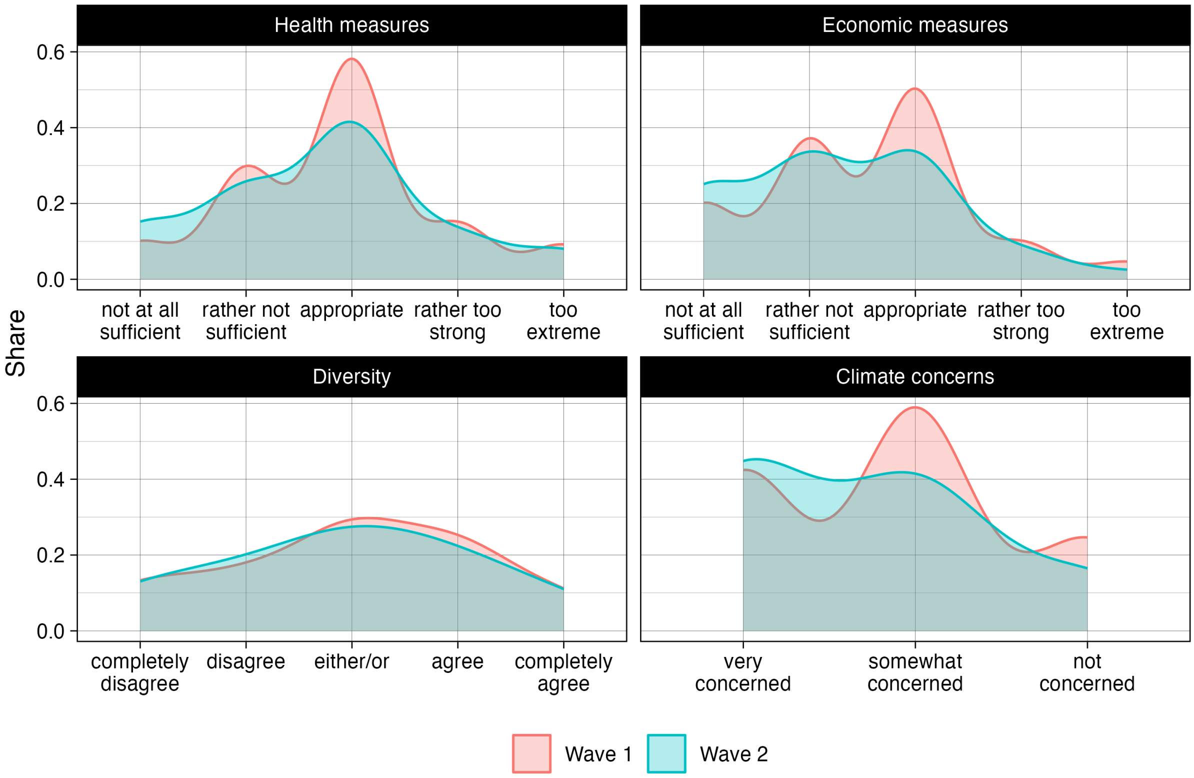

Other tables and figures referenced in the main text

Table 1: Individual-level differences in participation in noninstitutional forms

	Participation	Regular Participation
Intercept	0.41 (0.42)	-2.03 (0.60)***
Insiders - outsiders		
Trust in state	-0.15 (0.04)***	-0.13 (0.06)*
Sq(L-R scale)	0.03 (0.01)***	0.04 (0.01)***
Dominant party	-0.09 (0.08)	-0.34 (0.12)**
Organisational membership	0.46 (0.05)***	0.42 (0.05)***
Pol. controls		
Political interest	0.53 (0.05)***	0.70 (0.07)***
L-R scale	-0.05 (0.02)*	0.07 (0.03)**
Socio-demographics		
Age	-0.07 (0.02)***	-0.05 (0.03)*
Sq(Age)	0.00 (0.00)*	0.00 (0.00)
High education	-0.03 (0.09)	0.16 (0.13)
Female	0.08 (0.08)	0.07 (0.12)
Income	0.08 (0.05)	-0.01 (0.08)
Mid. town (ref: big city)	-0.13 (0.10)	0.16 (0.14)
Small town	-0.26 (0.10)*	-0.34 (0.16)*
Countryside	-0.30 (0.11)**	-0.27 (0.16)
McFadden Sq(R)	0.09	0.14
AIC	4115.83	2248.42
BIC	4207.47	2340.06
Num. obs.	3325	3325

$^{***}p < 0.001$; $^{**}p < 0.01$; $^{*}p < 0.05$. Results are based on the first wave, weighted by the socio-demographic weight.

Figure 5: LCA model fit with various classes

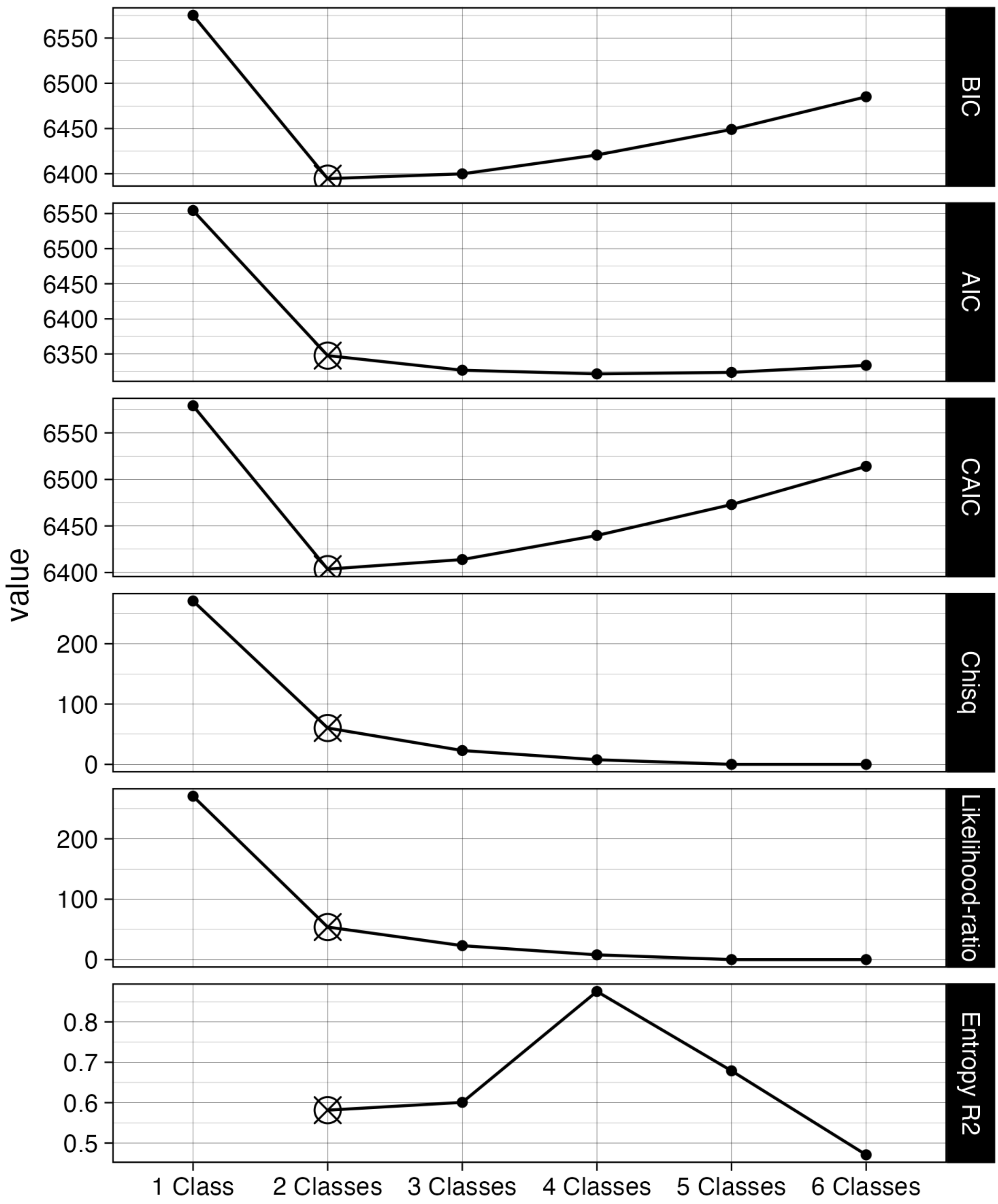

Table 2: Logistic Coefficient Estimates for Latent Class Analysis

	Model 1		Model 2	
	**Coef**	**SE**	**Coef**	**SE**
Intercept	-0.04**	(0.01)	-0.01	(0.02)
**Insiders - outsiders**				
Trust in state	-0.83***	(0.19)	-0.85***	(0.18)
Sq(L-R scale)	-0.07**	(0.03)	-0.05*	(0.02)
Org. Membership	-0.4**	(0.15)	-0.41**	(0.15)
Dominant party	-1.54***	(0.31)		
**Parties (ref: CDU/CSU)**				
NA/DK			1.17***	(0.33)
SPD			-1.35***	(0.25)
B90/Grüne			-0.95***	(0.05)
Linke			0.76*	(0.34)
FDP			0.01	(0.24)
AfD			0.82*	(0.38)
Other party			-0.19**	(0.06)
**Issue preferences**				
Climate concerns	-1.68***	(0.26)	-1.69***	(0.25)
Diversity	-0.56***	(0.15)	-0.61***	(0.15)
Health measures	0.87***	(0.17)	0.80***	(0.17)
Economic measures	0.39**	(0.15)	0.35*	(0.15)
**Forms of participation**				
Public demo.	0.29	(0.27)	0.35	(0.27)
Online demo.	0.05	(0.21)	-0.04	(0.21)
Posting online	0.13	(0.16)	0.18	(0.16)
Petitions	-0.58***	(0.17)	-0.51**	(0.16)
Illegal demo.	-0.2	(0.20)	-0.18	(0.21)
**Socio-demographics and controls**				
Political interest	0.36	(0.19)	0.32	(0.20)
L-R scale	0.48***	(0.10)	0.36***	(0.09)
Age	0.16***	(0.05)	0.12**	(0.05)
Sq(Age)	0.01**	(0.01)	0.01*	(0.01)
High educ.	0.40	(0.30)	0.36	(0.30)
Female	0.27	(0.30)	0.29	(0.30)
Income	0.23	(0.18)	0.24	(0.18)
Mid. town (ref: big city)	-0.48	(0.26)	-0.43	(0.28)
Small town	0.20	(0.26)	0.30	(0.26)
Countryside	0.30	(0.25)	0.44	(0.27)

$^{***}p < 0.001$; $^{**}p < 0.01$; $^{*}p < 0.05$

Table 3: Fixed effects model of issue specific participation

	Climate Protection	Against Racism	COVID-19 Economy	COVID-19 Freedom
Issue positions				
Climate worries	0.24 (0.37)	−1.23 (0.51)*	−0.08 (0.49)	0.73 (0.43)
Diversity	0.65 (0.29)*	−0.23 (0.36)	0.39 (0.30)	−0.15 (0.33)
Health measures	−0.62 (0.31)	−0.63 (0.33)	−0.37 (0.28)	0.54 (0.27)*
Econ. measures	−0.39 (0.30)	−1.29 (0.34)***	0.88 (0.29)**	0.07 (0.25)
Attitudes (controls)				
Pol. interest	−0.43 (0.43)	−1.39 (0.55)*	−1.10 (0.63)	1.13 (0.59)
L-R position	−0.16 (0.21)	−0.06 (0.23)	0.61 (0.30)*	0.74 (0.29)*
Trust in state	0.20 (0.31)	−0.50 (0.40)	0.80 (0.50)	−0.34 (0.40)
Log Likelihood	−17619	−12642	−10378	−10028
Deviance	352.38	252.84	207.55	200.57
Num. obs.	264	202	168	158
Num. pers.	132	101	84	79

$^{***}p < 0.001$; $^{**}p < 0.01$; $^{*}p < 0.05$

Appendix B: Robustness checks

Normalization across noninstitutional forms of participation

taken part in a lawful public protest activity (i.e. demonstration, human chain)?

Table 1: Individual-level differences in participation in a specific form

	Participation	Regular Participation
Intercept	-1.28 (0.62)*	-3.97 (1.27)**
Insiders - outsiders		
Trust in state	-0.01 (0.06)	0.18 (0.12)
Sq(L-R scale)	0.04 (0.01)***	0.05 (0.02)**
Dominant party	-0.10 (0.12)	0.14 (0.26)
Organisational membership	0.44 (0.06)***	0.57 (0.10)***
Pol. controls		
Political interest	0.35 (0.07)***	0.73 (0.16)***
L-R scale	0.12 (0.03)***	0.20 (0.05)***
Socio-demographics		
Age	-0.00 (0.03)	-0.04 (0.06)
Sq(Age)	-0.00 (0.00)*	-0.00 (0.00)
High education	-0.29 (0.13)*	-0.38 (0.26)
Female	-0.33 (0.12)**	-0.26 (0.26)
Income	-0.19 (0.08)*	-0.43 (0.18)*
Mid. town (ref: big city)	0.28 (0.15)	0.09 (0.27)
Small town	-0.15 (0.16)	-1.31 (0.42)**
Countryside	-0.43 (0.18)*	-1.06 (0.41)**
McFadden Sq(R)	0.15	0.22
AIC	2128.60	666.28
BIC	2220.21	757.89
Num. obs.	3319	3319

$^{***}p < 0.001$; $^{**}p < 0.01$; $^{*}p < 0.05$. Results are based on the first wave, weighted by the socio-demographic weight.

Figure 1: Individual-level differences in participation in noninstitutional forms

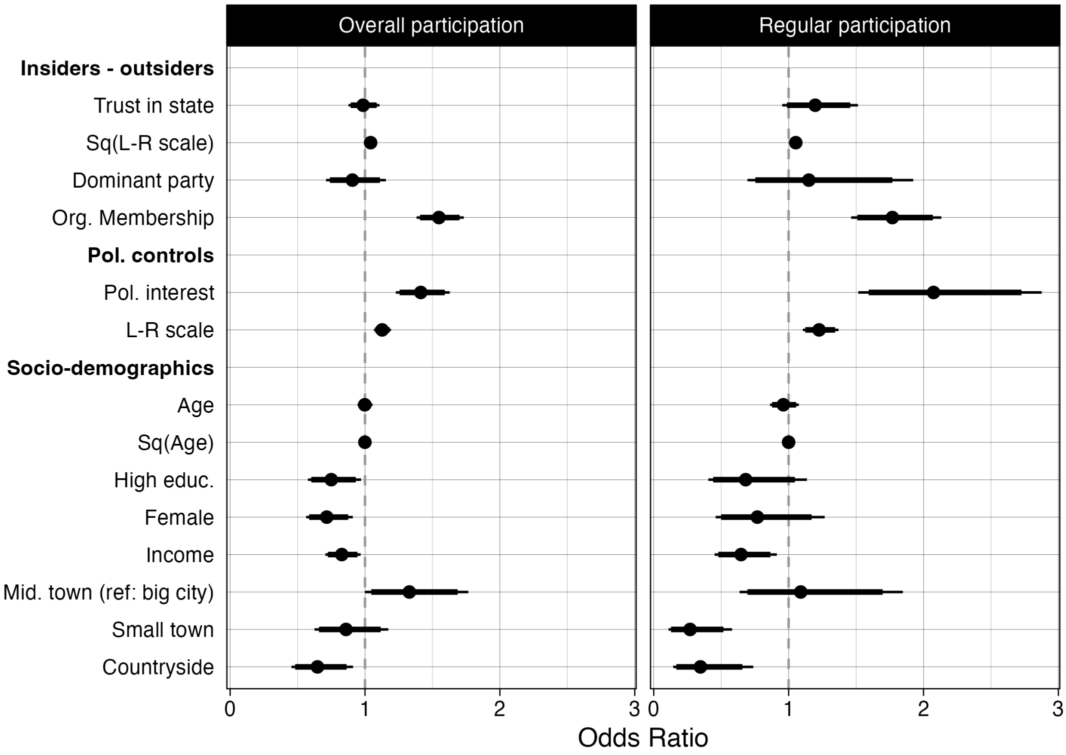

Note: Coefficients are presented as odds ratios. Results are based on the first wave, weighted by the socio-demographic weight.

taken part in an illegal public protest activity (i.e. demonstration, blockade)

Table 2: Individual-level differences in participation in a specific form

	Participation	Regular Participation
Intercept	−2.99 (0.74)***	−5.76 (1.20)***
Insiders - outsiders		
Trust in state	0.15 (0.07)*	0.45 (0.11)***
Sq(L-R scale)	0.04 (0.01)***	0.04 (0.02)*
Dominant party	0.06 (0.14)	0.25 (0.22)
Organisational membership	0.49 (0.06)***	0.64 (0.09)***
Pol. controls		
Political interest	0.18 (0.08)*	0.27 (0.13)*
L-R scale	0.17 (0.03)***	0.31 (0.06)***
Socio-demographics		
Age	0.08 (0.04)*	0.11 (0.06)*
Sq(Age)	−0.00 (0.00)***	−0.00 (0.00)**
High education	−0.47 (0.15)**	−0.37 (0.23)
Female	−0.65 (0.14)***	−0.46 (0.22)*
Income	−0.11 (0.09)	−0.35 (0.15)*
Mid. town (ref: big city)	0.40 (0.16)*	0.37 (0.24)
Small town	−0.31 (0.19)	−0.60 (0.31)
Countryside	−0.45 (0.21)*	−0.94 (0.36)**
McFadden Sq(R)	0.19	0.27
AIC	1658.12	812.80
BIC	1749.73	904.41
Num. obs.	3319	3319

***$p < 0.001$; **$p < 0.01$; *$p < 0.05$. Results are based on the first wave, weighted by the socio-demographic weight.

Figure 2: Individual-level differences in participation in noninstitutional forms

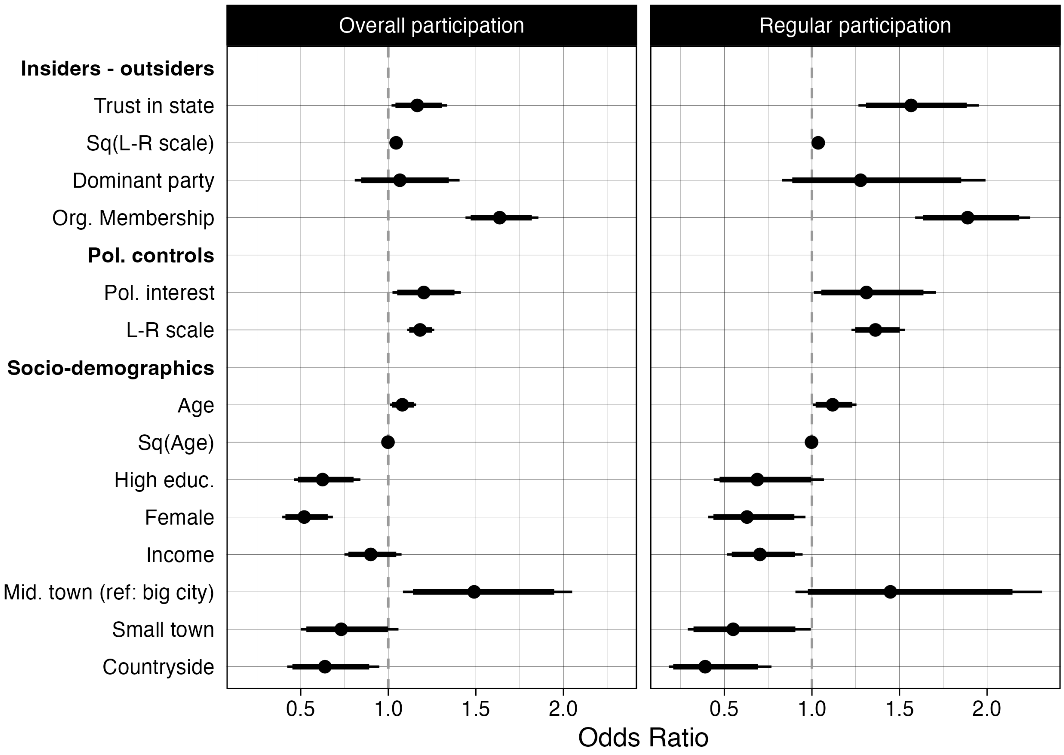

Note: Coefficients are presented as odds ratios. Results are based on the first wave, weighted by the socio-demographic weight.

taken part in a protest activity on the Internet (i.e. digital protest)?

Table 3: Individual-level differences in participation in a specific form

	Participation	Regular Participation
Intercept	−1.06 (0.57)	−5.52 (1.21)***
Insiders - outsiders		
Trust in state	−0.06 (0.06)	0.15 (0.11)
Sq(L-R scale)	0.03 (0.01)***	0.05 (0.01)**
Dominant party	−0.06 (0.12)	0.07 (0.24)
Organisational membership	0.41 (0.05)***	0.43 (0.09)***
Pol. controls		
Political interest	0.35 (0.07)***	0.72 (0.15)***
L-R scale	0.08 (0.03)**	0.12 (0.05)*
Socio-demographics		
Age	−0.03 (0.03)	0.01 (0.05)
Sq(Age)	−0.00 (0.00)	−0.00 (0.00)
High education	−0.07 (0.12)	0.14 (0.24)
Female	−0.28 (0.12)*	0.03 (0.23)
Income	−0.02 (0.07)	−0.12 (0.15)
Mid. town (ref: big city)	0.31 (0.14)*	−0.22 (0.27)
Small town	−0.31 (0.15)*	−0.78 (0.33)*
Countryside	−0.26 (0.16)	−0.78 (0.35)*
McFadden Sq(R)	0.11	0.15
AIC	2392.74	797.98
BIC	2484.35	889.59
Num. obs.	3319	3319

***$p < 0.001$; **$p < 0.01$; *$p < 0.05$. Results are based on the first wave, weighted by the socio-demographic weight.

Figure 3: Individual-level differences in participation in noninstitutional forms

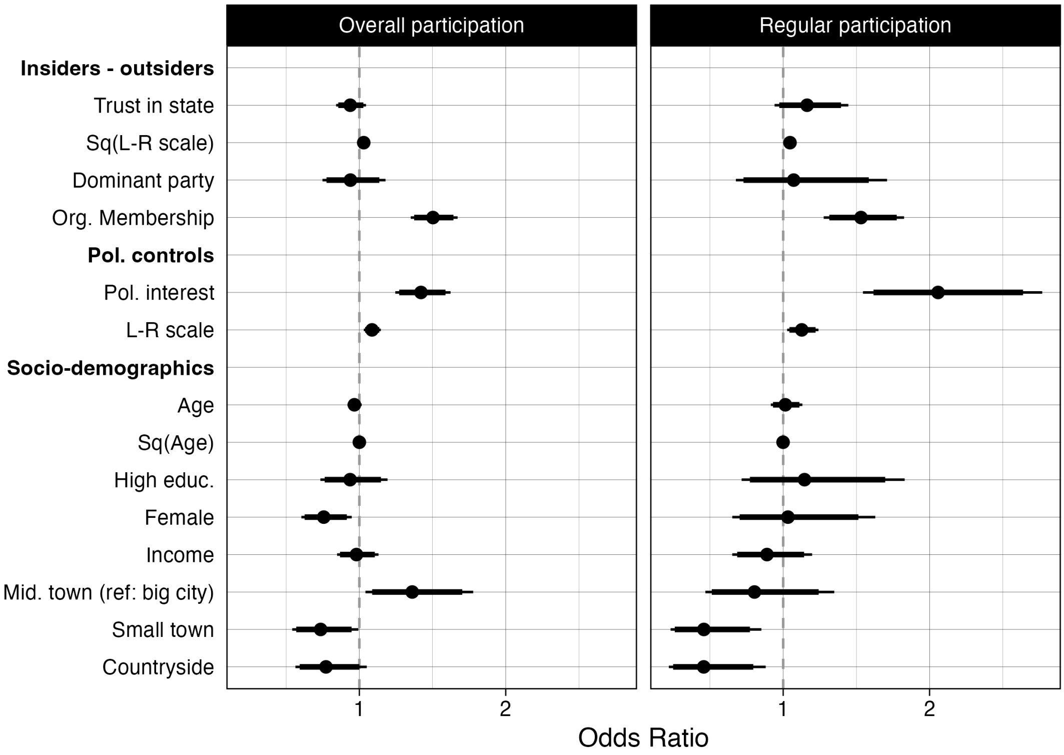

Note: Coefficients are presented as odds ratios. Results are based on the first wave, weighted by the socio-demographic weight.

posted about politics online, for example on blogs, via email or on social media such as Facebook or Twitter?

Table 4: Individual-level differences in participation in a specific form

	Participation	Regular Participation
Intercept	−0.39 (0.45)	−4.21 (0.82)***
Insiders - outsiders		
Trust in state	−0.20 (0.04)***	−0.13 (0.07)
Sq(L-R scale)	0.03 (0.01)***	0.05 (0.01)***
Dominant party	−0.09 (0.09)	−0.36 (0.16)*
Organisational membership	0.35 (0.05)***	0.27 (0.07)***
Pol. controls		
Political interest	0.49 (0.05)***	0.90 (0.10)***
L-R scale	−0.00 (0.02)	0.06 (0.03)
Socio-demographics		
Age	−0.04 (0.02)	−0.00 (0.04)
Sq(Age)	0.00 (0.00)	−0.00 (0.00)
High education	−0.02 (0.10)	0.05 (0.17)
Female	−0.07 (0.09)	0.17 (0.16)
Income	0.04 (0.06)	−0.06 (0.10)
Mid. town (ref: big city)	0.01 (0.11)	0.22 (0.19)
Small town	−0.24 (0.11)*	−0.45 (0.22)*
Countryside	−0.23 (0.12)	−0.21 (0.22)
McFadden Sq(R)	0.09	0.13
AIC	3626.32	1446.38
BIC	3717.93	1537.99
Num. obs.	3319	3319

***$p < 0.001$; **$p < 0.01$; *$p < 0.05$. Results are based on the first wave, weighted by the socio-demographic weight.

Figure 4: Individual-level differences in participation in noninstitutional forms

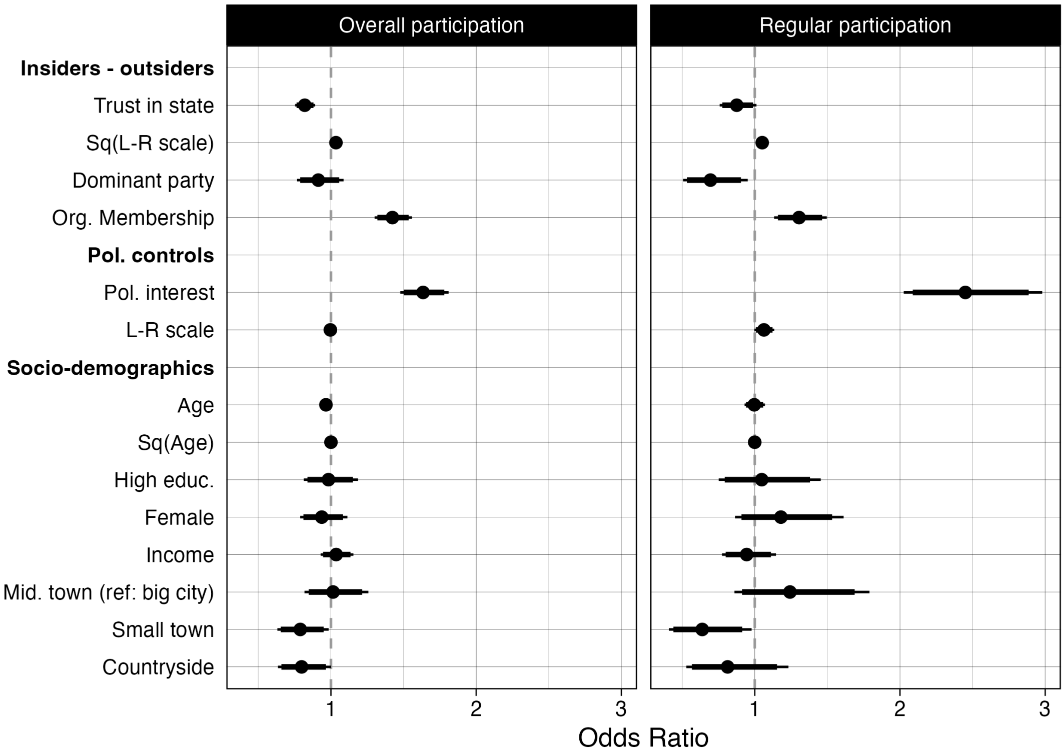

Note: Coefficients are presented as odds ratios. Results are based on the first wave, weighted by the socio-demographic weight.

signed a petition?

Table 5: Individual-level differences in participation in a specific form

	Participation	Regular Participation
Intercept	−0.18 (0.44)	−2.91 (0.73)***
Insiders - outsiders		
Trust in state	−0.12 (0.04)**	−0.08 (0.07)
Sq(L-R scale)	0.02 (0.01)**	0.04 (0.01)***
Dominant party	−0.10 (0.09)	−0.31 (0.15)*
Organisational membership	0.43 (0.05)***	0.44 (0.06)***
Pol. controls		
Political interest	0.55 (0.05)***	0.74 (0.09)***
L-R scale	−0.07 (0.02)**	0.04 (0.03)
Socio-demographics		
Age	−0.07 (0.02)***	−0.06 (0.03)*
Sq(Age)	0.00 (0.00)*	0.00 (0.00)
High education	−0.04 (0.09)	0.10 (0.15)
Female	0.12 (0.09)	0.37 (0.15)*
Income	0.09 (0.05)	0.02 (0.09)
Mid. town (ref: big city)	−0.08 (0.11)	−0.08 (0.18)
Small town	−0.33 (0.11)**	−0.36 (0.19)
Countryside	−0.23 (0.11)*	−0.31 (0.20)
McFadden Sq(R)	0.09	0.12
AIC	3759.98	1669.32
BIC	3851.59	1760.93
Num. obs.	3319	3319

$^{***}p < 0.001$; $^{**}p < 0.01$; $^{*}p < 0.05$. Results are based on the first wave, weighted by the socio-demographic weight.

Figure 5: Individual-level differences in participation in noninstitutional forms

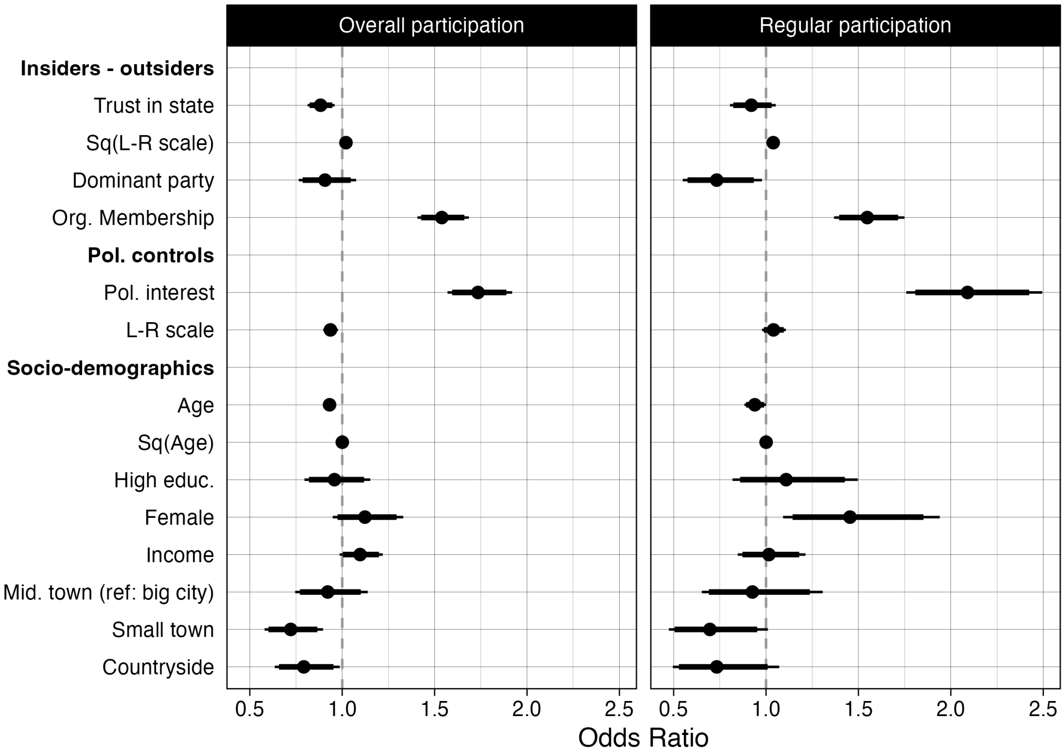

Note: Coefficients are presented as odds ratios. Results are based on the first wave, weighted by the socio-demographic weight.

Logit replication of the LCA model

Table 6: Logit models: issue specific participation

	Climate	Against rac.	Cov. Econ.	Cov. Freed.
Intercept	−3.02 (0.89)***	−1.78 (0.90)*	−3.70 (0.94)***	−5.84 (1.08)***
Insiders - outsiders				
Trust in state	0.19 (0.08)*	0.09 (0.08)	0.03 (0.08)	−0.35 (0.09)***
Sq(L-R scale)	−0.02 (0.01)	0.03 (0.01)*	−0.00 (0.01)	−0.02 (0.01)
Org. Membership	0.16 (0.07)*	0.23 (0.07)**	0.21 (0.07)**	0.17 (0.08)*
Parties (ref: CDU/CSU)				
NA/DK	−0.65 (0.23)**	−0.25 (0.24)	−0.02 (0.24)	0.61 (0.27)*
SPD	−0.36 (0.24)	0.72 (0.24)**	−0.31 (0.27)	−0.21 (0.34)
B90/Grüne	0.46 (0.23)*	−0.05 (0.23)	0.07 (0.25)	−0.24 (0.35)
Linke	−0.12 (0.27)	−0.10 (0.28)	0.02 (0.30)	0.46 (0.35)
FDP	−0.67 (0.35)	0.46 (0.33)	0.39 (0.33)	0.44 (0.38)
AfD	−0.45 (0.28)	−1.11 (0.36)**	−0.61 (0.30)*	0.90 (0.30)**
Other party	−0.20 (0.30)	−0.08 (0.31)	−0.56 (0.35)	0.07 (0.39)
Issue preferences				
Climate concerns	1.01 (0.11)***	0.24 (0.11)*	−0.14 (0.11)	−0.24 (0.12)
Diversity	0.09 (0.07)	0.47 (0.07)***	−0.05 (0.07)	−0.09 (0.08)
Health measures	−0.24 (0.08)**	−0.02 (0.08)	0.08 (0.07)	0.68 (0.08)***
Econ. measures	−0.11 (0.08)	0.06 (0.08)	0.24 (0.07)***	0.16 (0.08)*
Forms of participation				
Public demo.	−0.21 (0.12)	−0.08 (0.13)	−0.18 (0.12)	0.15 (0.12)
Online demo.	0.11 (0.10)	0.12 (0.10)	0.23 (0.10)*	0.22 (0.11)*
Posting online	−0.03 (0.07)	0.39 (0.07)***	0.17 (0.07)*	0.43 (0.08)***
Petitions	0.31 (0.07)***	0.07 (0.07)	−0.03 (0.07)	−0.03 (0.08)
Illegal demo.	0.29 (0.10)**	−0.22 (0.11)*	−0.05 (0.11)	−0.05 (0.11)
Socio-demog. & controls				
Pol. interest	−0.09 (0.09)	−0.05 (0.10)	0.01 (0.10)	0.06 (0.11)
L-R scale	−0.02 (0.04)	−0.17 (0.05)***	0.03 (0.05)	0.07 (0.05)
Age	0.00 (0.03)	−0.10 (0.03)***	0.05 (0.03)	0.08 (0.04)
Sq(Age)	−0.00 (0.00)	0.00 (0.00)**	−0.00 (0.00)	−0.00 (0.00)
High educ.	0.11 (0.16)	−0.45 (0.16)**	−0.02 (0.17)	−0.01 (0.19)
Female	−0.21 (0.14)	0.09 (0.15)	0.26 (0.15)	−0.20 (0.18)
Income	−0.04 (0.09)	−0.10 (0.09)	0.02 (0.09)	0.11 (0.11)
Mid. town (ref: big c.)	0.40 (0.18)*	0.27 (0.18)	−0.03 (0.18)	0.16 (0.21)
Small town	0.22 (0.18)	−0.23 (0.19)	−0.37 (0.20)	0.15 (0.22)
Countryside	−0.01 (0.19)	−0.24 (0.20)	−0.03 (0.20)	−0.02 (0.23)
Part. on other iss.				
Climate prot.		0.51 (0.15)***	−0.28 (0.16)	−0.47 (0.19)*
Against racism	0.51 (0.15)***		0.22 (0.16)	−0.20 (0.20)
Corona econ.	−0.26 (0.17)	0.22 (0.17)		−0.06 (0.19)
Corona freedom	−0.55 (0.19)**	−0.32 (0.20)	−0.07 (0.19)	
McFadden Sq(R)	0.24	0.23	0.05	0.31
AIC	1488.56	1440.56	1408.35	1085.40
BIC	1660.72	1612.71	1580.50	1257.55
Num. obs.	1362	1362	1362	1362

***$p < 0.001$; **$p < 0.01$; *$p < 0.05$. Results are based on the first wave, weighted by the socio-demographic weight.

Figure 6: Issue specific participation

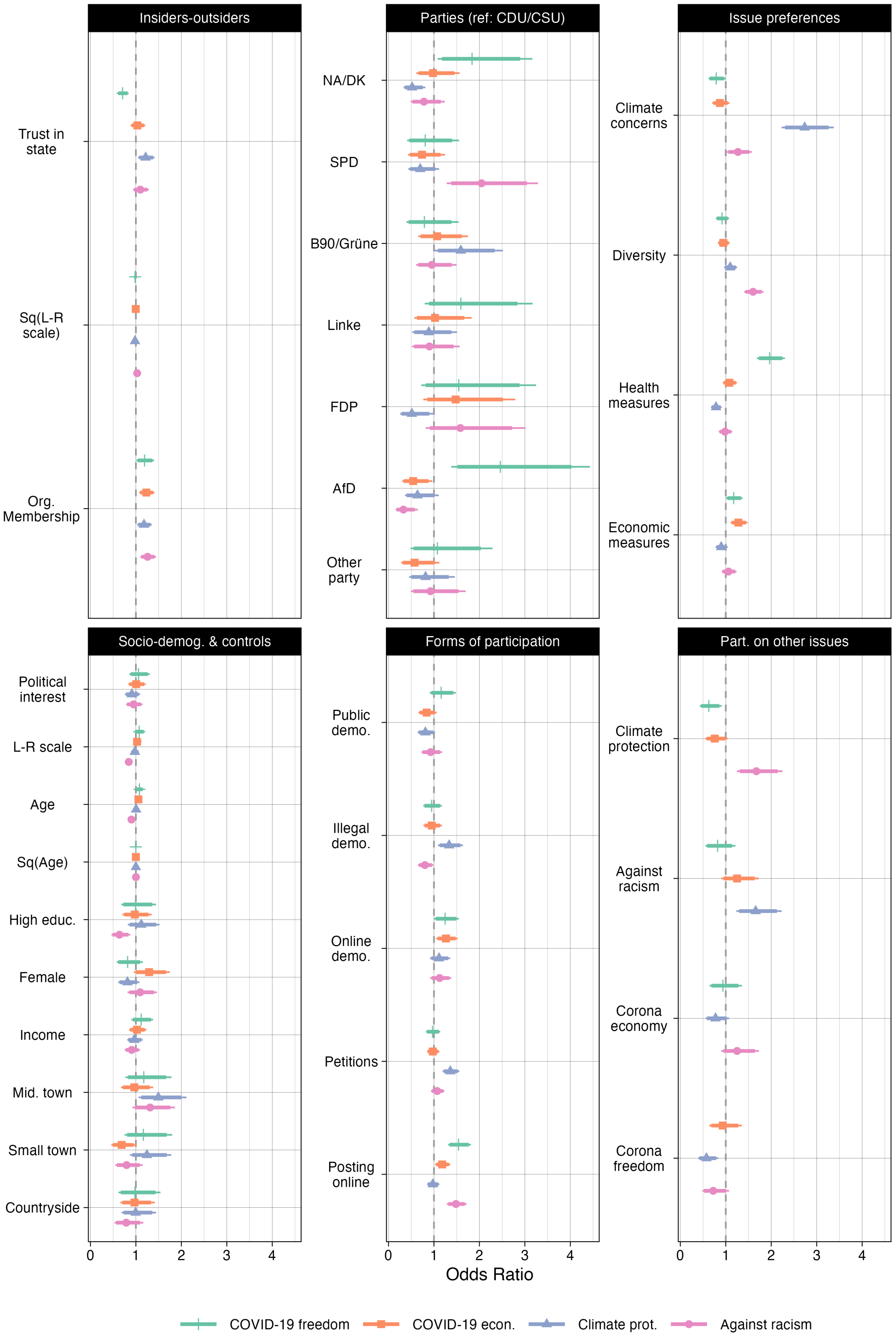

Note: Coefficients are presented as odds ratios. See the full regression table in Appendix B, table 6. The thicker error bars represent 90 percent confidence intervals, the thinner error bars represent 95 percent confidence intervals. Results are based on the first wave, weighted by the socio-demographic weight.

The effect of differentiated partisanship on participation

Table 7: Individual-level differences in participation in noninstitutional forms

	Participation	Regular Participation
Intercept	0.09 (0.44)	−2.66 (0.63)***
Insiders - outsiders		
Trust in state	−0.12 (0.04)**	−0.11 (0.06)
Sq(L-R scale)	0.02 (0.01)*	0.03 (0.01)***
Organisational membership	0.46 (0.05)***	0.41 (0.06)***
Parties (ref: CDU/CSU)		
NA/DK	0.08 (0.12)	0.31 (0.21)
SPD	0.31 (0.14)*	0.37 (0.23)
B90/Grüne	0.42 (0.14)**	0.61 (0.21)**
Linke	0.49 (0.17)**	0.94 (0.25)***
FDP	0.36 (0.20)	0.68 (0.28)*
AfD	0.70 (0.15)***	0.90 (0.22)***
Other party	0.30 (0.18)	0.91 (0.26)***
Pol. controls		
Political interest	0.48 (0.05)***	0.65 (0.08)***
L-R scale	−0.05 (0.02)*	0.09 (0.03)**
Socio-demographics		
Age	−0.06 (0.02)***	−0.05 (0.03)
Sq(Age)	0.00 (0.00)	0.00 (0.00)
High education	−0.06 (0.09)	0.12 (0.13)
Female	0.11 (0.08)	0.10 (0.12)
Income	0.08 (0.05)	−0.01 (0.08)
Mid. town (ref: big city)	−0.13 (0.10)	0.14 (0.14)
Small town	−0.26 (0.10)*	−0.34 (0.16)*
Countryside	−0.29 (0.11)**	−0.27 (0.16)
McFadden Sq(R)	0.10	0.15
AIC	4090.66	2234.44
BIC	4218.96	2362.73
Num. obs.	3325	3325

***$p < 0.001$; **$p < 0.01$; *$p < 0.05$. Results are based on the first wave, weighted by the socio-demographic weight.

Figure 7: Participation in noninstitutional forms with differentiated partisanship

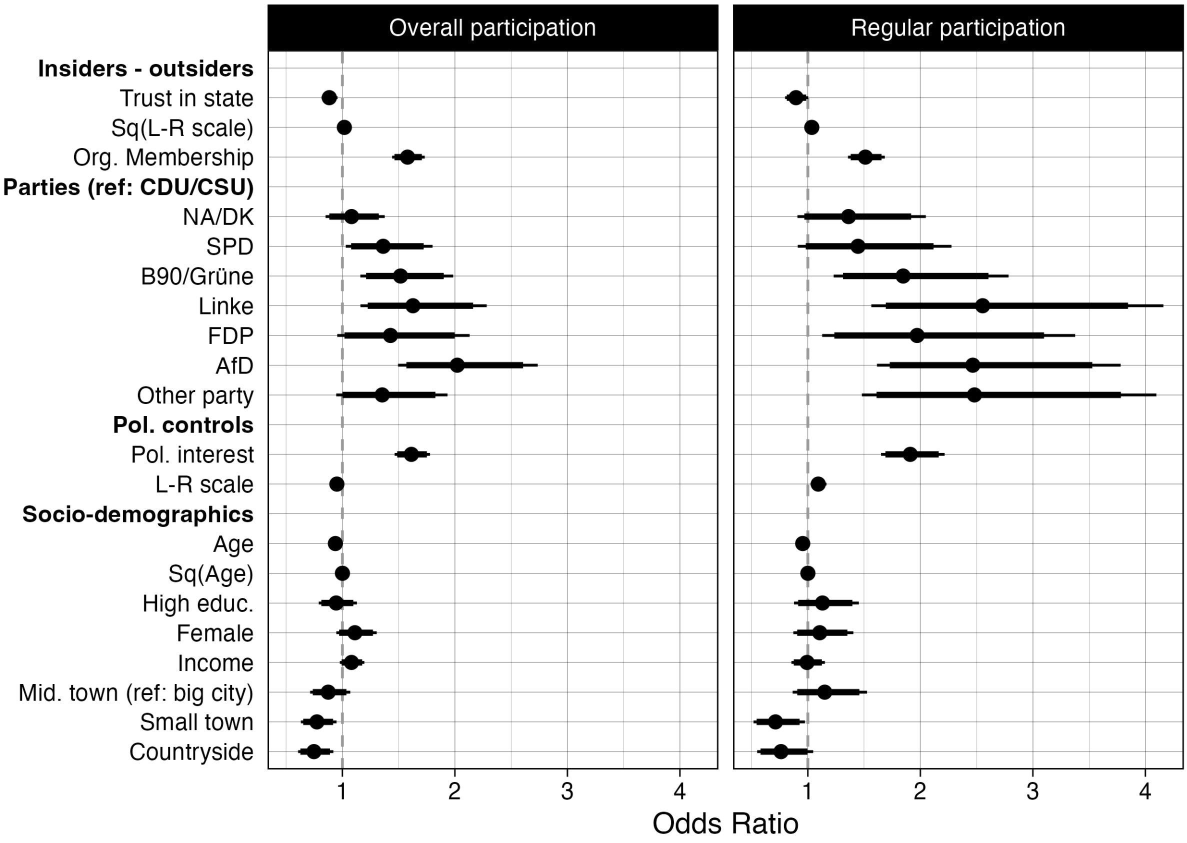

Note: See the corresponding regression model included above, table 7, Appendix B. The thicker error bars represent 90 percent confidence intervals, the thinner error bars represent 95 percent confidence intervals. Results are based on the first wave, weighted by the socio-demographic weight.

Replication with differentiated ideological scales

In this part of the analysis the left-right scale is exchanged with an economic and a cultural left-right ideological scale. For the economic scale, I rely on the responses to the item 'The state should take measures to reduce income inequality'. For the cultural scale, I rely on the responses to the item 'Germany is becoming a worse place to live because of immigration.' Both are measured on a five points scale (see appendix C). I reversed the economic scale, so that positive values represent more right-wing positions.

Table 8: Individual-level differences in participation in noninstitutional forms

	Participation	Regular Participation
Intercept	0.33 (0.43)	−2.05 (0.60)***
Insiders - outsiders		
Trust in state	−0.16 (0.04)***	−0.13 (0.06)*
Sq(gov. intervention)	−0.00 (0.03)	0.02 (0.04)
Sq(migration)	0.03 (0.02)	0.03 (0.03)
Organisational membership	0.46 (0.05)***	0.43 (0.05)***
Dominant party	−0.15 (0.08)	−0.39 (0.12)**
Pol. controls		
Political interest	0.54 (0.05)***	0.73 (0.07)***
Gov. intervention	0.04 (0.05)	0.00 (0.07)
Migration	−0.07 (0.03)*	0.03 (0.04)
Socio-demographics		
Age	−0.06 (0.02)***	−0.04 (0.03)
Sq(Age)	0.00 (0.00)*	0.00 (0.00)
High education	−0.06 (0.09)	0.11 (0.13)
Female	0.09 (0.08)	0.05 (0.12)
Income	0.10 (0.05)	−0.02 (0.08)
Mid. town (ref: big city)	−0.14 (0.10)	0.14 (0.14)
Small town	−0.29 (0.10)**	−0.36 (0.16)*
Countryside	−0.32 (0.11)**	−0.30 (0.16)
McFadden Sq(R)	0.09	0.13
AIC	4136.14	2288.09
BIC	4240.01	2391.96
Num. obs.	3327	3327

***$p < 0.001$; **$p < 0.01$; *$p < 0.05$. Results are based on the first wave, weighted by the socio-demographic weight.

Figure 8: Participation in noninstitutional forms with differentiated ideological scales

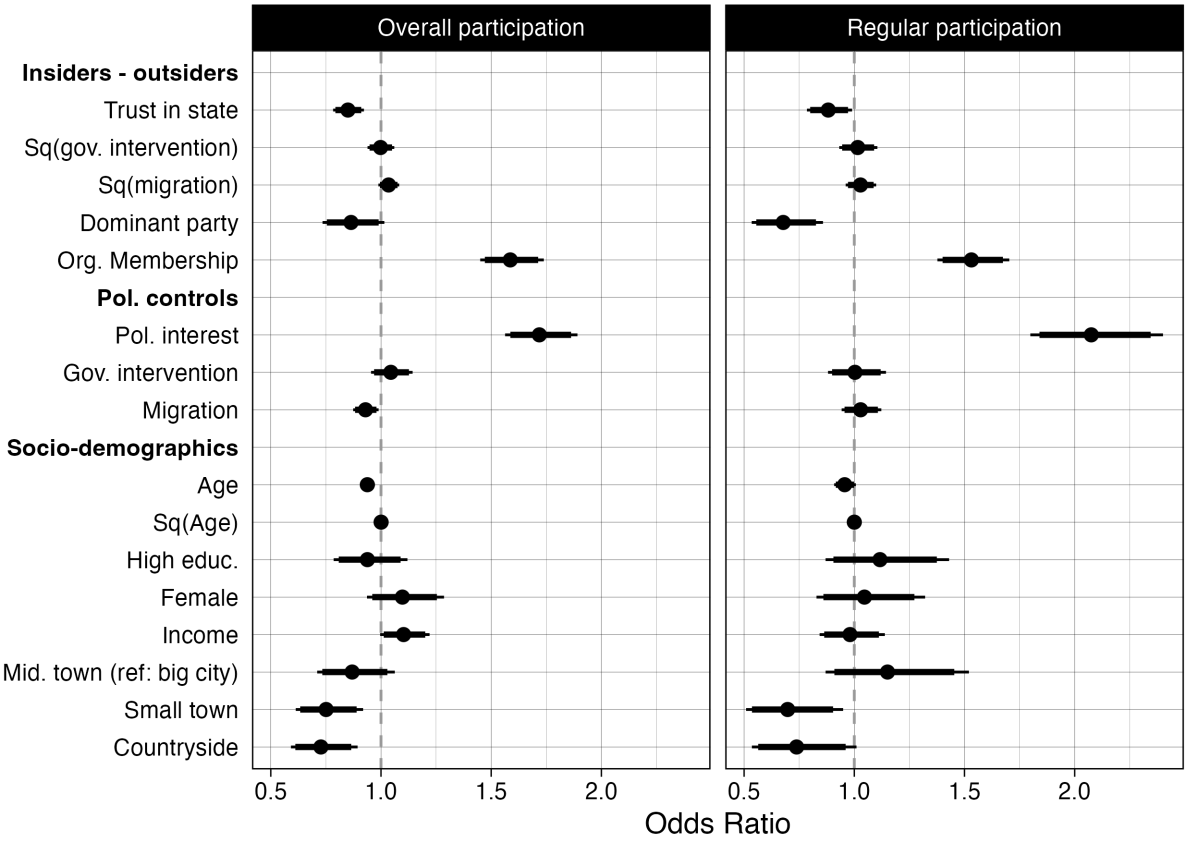

Note: See the corresponding regression model included above, table 8, Appendix B. The thicker error bars represent 90 percent confidence intervals, the thinner error bars represent 95 percent confidence intervals. Results are based on the first wave, weighted by the socio-demographic weight.

Table 9: Logistic Coefficient Estimates for Latent Class Analysis

	Model 1		Model 2	
	**Coef**	**SE**	**Coef**	**SE**
Intercept	-0.04***	(0.01)	-0.01	(0.02)
**Insiders - outsiders**				
Trust in state	-0.77***	(0.18)	-0.78***	(0.17)
Sq(gov. intervention)	0.01	(0.13)	0.01	(0.13)
Sq(migration)	-0.02	(0.11)	-0.02	(0.11)
Org. Membership	-0.51***	(0.15)	-0.5***	(0.15)
Dominant party	-1.48***	(0.31)		
**Parties (ref: CDU/CSU)**				
NA/DK			1.33***	(0.31)
SPD			-1.05**	(0.41)
B90/Grüne			-0.98***	(0.11)
Linke			0.38	(0.36)
FDP			-0.17	(0.36)
AfD			1.17***	(0.34)
Other party			-0.33***	(0.07)
**Issue preferences**				
Climate concerns	-1.53***	(0.25)	-1.51***	(0.23)
Diversity	-0.5**	(0.18)	-0.49**	(0.18)
Health measures	0.80***	(0.17)	0.70***	(0.17)
Economic measures	0.38*	(0.15)	0.33*	(0.15)
**Forms of participation**				
Public demo.	0.30	(0.25)	0.37	(0.26)
Online demo.	0.01	(0.21)	-0.04	(0.22)
Posting online	0.04	(0.15)	0.05	(0.16)
Petitions	-0.55***	(0.16)	-0.53***	(0.16)
Illegal demo.	-0.14	(0.19)	-0.15	(0.19)
**Socio-demographics and controls**				
Political interest	0.37	(0.19)	0.41*	(0.20)
Gov. intervention	0.58**	(0.19)	0.46*	(0.19)
Migration	0.67***	(0.17)	0.57***	(0.17)
Age	0.14**	(0.05)	0.10*	(0.05)
Sq(Age)	0.01*	(0.01)	0.01	(0.01)
High educ.	0.35	(0.30)	0.41	(0.29)
Female	0.26	(0.29)	0.27	(0.29)
Income	0.18	(0.18)	0.17	(0.18)
Mid. town (ref: big city)	-0.53*	(0.25)	-0.47	(0.28)
Small town	0.12	(0.25)	0.21	(0.26)
Countryside	0.40	(0.25)	0.55*	(0.28)

$^{***}p < 0.001$; $^{**}p < 0.01$; $^{*}p < 0.05$

Figure 9: Latent class analysis with differentiated ideological scales

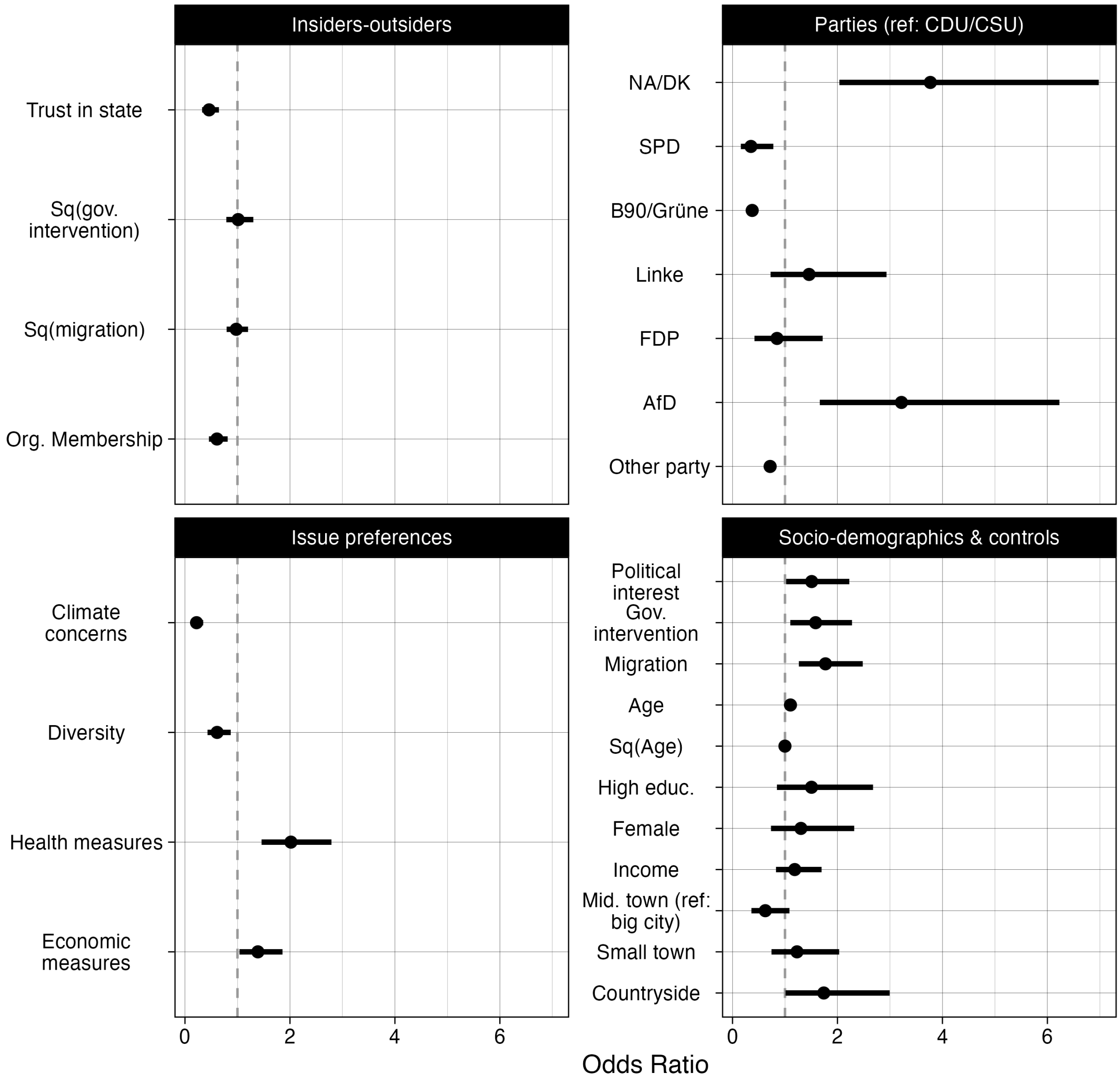

The model predict membership in latent classes with covariates. It also include controls for the form of engagement. See the full regression above, table 9, appendix B. The error bars represent 95 percent confidence intervals.

Appendix C: Survey items

Dependent variables

We are initially interested in societal engagement in the time since public life was restricted in many European countries in March due to the Corona Pandemic. This posed a great challenge for many people and made traditional forms of engagement difficult. [Wir interessieren uns zunächst für soziales Engagement in der Zeit seitdem in vielen europäischen Ländern im März das öffentliche Leben aufgrund der Corona-Pandemie eingeschränkt wurde. Dies stellte eine große Herausforderung für viele Menschen dar und erschwerte herkömmliche Formen des Engagements.]

[Civic engagement block]

There are other possibilities to engage socially or politically. Since the beginning of the corona crisis until today: How often did you...
[Es gibt noch weitere Möglichkeiten, sich gesellschaftlich oder politisch zu engagieren. Seit Beginn der Corona-Krise bis heute: Wie oft haben Sie ...?]

	**Never [Nie]**	**Rarely [Selten]**	**Sometimes [Manchmal]**	**Often [Oft]**	**Very often [Sehr oft]**
taken part in a lawful public protest activity (i.e. demonstration, human chain)? [an einer genehmigten Protestaktion im öffentlichen Raum teilgenommen (z.B. Demonstration, Menschenkette)?]					
taken part in an illegal public protest activity (i.e. demonstration, blockade)? [an einer nicht genehmigten Protestaktion im öffentlichen Raum teilgenommen (z.B. Demonstration, Blockade)?]					
taken part in a protest activity on the Internet (i.e. digital protest)? [an einer Protestaktion im Internet teilgenommen (z.B. Netzstreik)?]					
posted about politics online, for example on blogs, via email or on social media such as Facebook or Twitter? [politische Inhalte Im Internet gepostet oder geteilt, zum Beispiel auf Blogs, per E-Mail oder in sozialen Medien wie Facebook oder Twitter?]					
signed a petition? [sich an einer Petition oder Unterschriftensammlung beteiligt?]					
contacted a politician, government or local government official? [Kontakt zu einer*einem Politiker*in oder einer Amtsperson auf Bundes-, Landes- oder Kommunalebene aufgenommen?]					
taken part in activities of political parties? [sich an Aktivitäten politischer Parteien beteiligt?]					
other forms of political participation? sich in anderer Form politisch beteiligt?					

You have indicated that you have been politically engaged since the beginning of the Corona Pandemic. On what issue have you been engaged?
[Sie haben angegeben, sich seit Beginn der Corona-Pandemie politisch engagiert zu haben. Zu welchem Thema haben Sie sich engagiert?]

against racism [Gegen Rassismus]	☐
for climate protection [Für Klimaschutz]	☐
against limitations to freedom due to the corona crisis [Gegen Freiheitseinschränkungen aufgrund der Corona-Krise]	☐
for governmental economic help related to the corona crisis [Für staatliche Wirtschaftshilfen aufgrund der Corona-Krise]	☐
other issues [Andere Forderungen]	☐

Independent variables

Overall, how do you rate the political measures... [Wie beurteilen Sie insgesamt die von der Politik ergriffenen Maßnahmen...]

	Not at all sufficient [überhaupt nicht ausreichend]	rather not sufficient [eher nicht ausreichend]	appropriate [angemessen]	rather too strong [eher zu stark]	too extreme [zu extrem]
...to address the health risks of the Corona crisis? [...zur Bewältigung der gesundheitlichen Risiken der Corona-Krise?]					
...to deal with the economic consequences of the Corona crisis? [...zur Bewältigung der wirtschaftlichen Folgen der Corona-Krise?]					

How about the following areas - are you concerned about them? [Wie ist es mit den folgenden Gebieten – machen Sie sich da Sorgen?]

	Very concerned [Große Sorgen]	Somewhat concerned [Einige Sorgen]	Not concerned [Keine Sorgen]	NA [KA]
To protect the environment [Um den Schutz der Umwelt]				

How much do you agree or disagree with the following statements? [Wie sehr stimmen Sie den folgenden Aussagen zu oder wie sehr lehnen Sie diese ab?]

	Completely disagree [Lehne stark ab]	Disagree [Lehne ab]	Either or [Weder noch]	Agree [Stimme zu]	Completely agree [Stimme stark zu]
Increasing diversity is threatening life in Germany in general [Durch zunehmende Vielfalt wird das Leben in Deutschland im Allgemeinen bedroht.]					

How interested are you in politics? Are you... [Wie sehr interessieren Sie sich für Politik? Sind Sie...]

not interested at all [überhaupt nicht interessiert]	little interested [wenig interessiert]	quite interested [ziemlich interessiert]	very interested [sehr interessiert]

In politics, people sometimes talk about ”left” and ”right”. Where on the scale would you classify yourself if 0 is left and 10 is right? [In der Politik spricht man manchmal von ”links” und ”rechts”. Wo auf der Skala würden Sie sich selbst einstufen, wenn 0 für links steht und 10 für rechts?]

Left [Links]										Right [Rechts]
0	1	2	3	4	5	6	7	8	9	10

How much do you agree or disagree with the following statements? [Wie sehr stimmen Sie den folgenden Aussagen zu oder wie sehr lehnen Sie diese ab?]

	Completely disagree [Lehne stark ab]	Disagree [Lehne ab]	Either or [Weder noch]	Agree [Stimme zu]	Completely agree [Stimme stark zu]
The state should take measures to reduce income inequality [Der Staat sollte Maßnahmen ergreifen, um Einkommensunterschiede zu verringern]					
Germany is becoming a worse place to live because of immigration. [Deutschland wird durch Zuwanderung zu einem schlechteren Ort zum Leben.]					

What is your view on the following statements? [Wie ist Ihre Meinung zu den folgenden Aussagen?]

	completely disagree [stimmt gar nicht]	mostly disagree [stimmt wenig]	So, so [teils, teils]	mostly agree [stimmt ziemlich]	completely agree [stimmt völlig]
In general, the state institutions in Germany can be trusted. [Im Allgemeinen kann man den staatlichen Institutionen in Deutschland vertrauen.]					

Are you a member of one or more of the following organisations? Please tick all that apply. [Sind Sie Mitglied in einer oder mehreren der folgenden Organisationen? Bitte kreuzen Sie alle zutreffenden Organisationen an.]

Trade union or professional association<br>[Gewerkschaft oder Berufsverband]	
Political party<br>[Politische Partei]	
Ecclesiastical or religious association<br>[Kirchliche oder religiöse Vereinigung]	
Sports club<br>[Sportverein]	
Leisure time organization (e.g. music, art)<br>[Freizeitorganisation (z.B. Musik, Kunst)]	
Environmental organisation or animal welfare association<br>[Umweltorganisation oder Tierschutzverein]	
Neighbourhood association<br>[Nachbarschaftsverein]	
Human rights organisation<br>[Menschenrechtsorganisation]	
Charity<br>[Wohlfahrtsorganisation]	
Patriotic Alliance<br>[Patriotisches Bündnis]	
In another civil society organisation<br>[In einer anderen zivilgesellschaftlichen Organisation]	
None of them<br>[Keine davon]	

Which party would you vote for if there were a federal election next Sunday?
[Welche Partei würden Sie wählen, wenn am nächsten Sonntag Bundestagswahl wäre?]

CDU/ CSU	
SPD	
Bündnis 90/ Die Grünen	
AfD	
Die Linke	
FDP	
Another party<br>[Andere Partei]	
Don't know<br>[Weiß nicht]	
I would not participate<br>[Ich würde nicht teilnehmen]	

How old are you? [Wie alt sind Sie?]

What is your gender? [Was ist Ihr Geschlecht?]

Male [Männlich]	
Female [Weiblich]	
Other [Divers]	

What is the highest general school-leaving qualification you have achieved? [Was ist der höchste allgemeinbildende Schulabschluss, den Sie erreicht haben?]

Grundschule nicht beendet	
(Noch) kein Schulabschluss, aber Grundschule beendet	
Abschluss einer Förderschule (Sonderschule, Hilfsschule)	
Volks- oder Hauptschule / Polytechn. Oberschule (8./9. Klasse)	
Mittlere Reife, Realschule / MSA / Polytechn. Oberschule (10. Klasse)	
Fachhochschulreife	
Abitur, fachgebundene Hochschulreife / Erweiterte Oberschule (12. Klasse)	
Zwischenprüfung, Vordiplom	
Diplom einer Berufsakademie (BA)	
Bachelor einer Verwaltungs-/Fachhochschule (FH), Berufsakademie	
Bachelor einer Universität	
Diplom einer Verwaltungs-/Fachhochschule (FH, auch frühere Ingenieurschule)	
Master einer Verwaltungs-/Fachhochschule (FH) (auch duale Hochschule BW)	
Master/Aufbaustudium Universität	
Diplom, M.A., 1. StEx Universität	
Promotion oder Habilitation	

How would you describe your current household income? With the current income I/we can... [Wie würden Sie Ihr gegenwärtiges Haushaltseinkommen beschreiben? Mit dem gegenwärtigen Einkommen kann ich/können wir...]

live comfortably [bequem leben]	
cope [zurechtkommen]	
find it difficult to cope [nur schwer zurechtkommen]	
very difficult to cope with [nur sehr schwer zurechtkommen]	

Which is most applicable to the residential area where you live? [Was trifft am ehesten auf das Wohngebiet zu, in dem Sie leben?](1)

Big city [Großstadt]	
Middle-sized city [Mittlere Stadt]	
Small town [Kleinstadt]	
Countryside [Auf dem Land]	

Appendix D: Survey weights

We construct two different types of weights: socio-demographic and nonresponse. The socio-demographic weight is applied for age, gender, education, region, and are based on official statistics from Eurostat for 2020. These weights are calculated using entropy balancing as implemented by the 'ebal' package in R (Hainmueller and Xu, 2013). Nonresponse weight is calculated, as described by Lohr (2019, p. 340-346), in three steps.

First, using a dichotomized version of all variables in the first wave of the survey, I set up a logistic regression model that predicts being included in the second wave. From these 381 independent variables in a stepwise fashion all are excluded that at a $p < 0.05$ threshold have no identifiable effect. The procedure leaves 32 dichotomized variables that have an identifiable effect on being included in the second wave. In a second step these are used to construct a logistic regression model, with the results presented by table 1.

As the table shows, the model only explains 6 percent of the variation, confirming that, at least in terms of their observable characteristics, individuals are to a great extent randomly included/ missing at random in the second wave of the survey. The two largest effects are for: 1. often contacting a politician in the year prior to the COVID-19 crisis (odds ratio = 1.72); and 2. having a centrist position on the question of being someone who can forgive (odds ratio = 0.56).

In a third step, I use the model to predict the inverse probability of being included in the second wave of data collection for each individual in the first wave. The inverse probability is then multiplied with the socio-demographic weight calculated for the first wave. The product of the two weights becomes the nonresponse weight for the second wave.

Table 2 shows the final set of weights that are used in the paper. As the table shows, with the quotas having been by and large met during both waves of the data collection, the two types of weight only implement minimal correction. The two largest corrections are implemented for education and region. The first wave of the data collection included roughly seven percent more highly educated individuals, and about five percent less eastern German respondents than Eurostat recorded. These two are the only deviations that are statistically significant at the $p < 0.05$ level.

References

Jens Hainmueller and Yiqing Xu. ebalance: A stata package for entropy balancing. *Journal of Statistical Software*, 54(7):1–18, 2013. URL `https://web.stanford.edu/~jhain/Paper/JSS2013.pdf`.

Sharon L. Lohr. *Sampling Design and Analysis (Second Edition).* CRC Press, Taylor & Francis Group, Boca Raton, London, New York, 2019. ISBN 978-0-3672-7346-0.

Table 1: Logistic regression model: Predicting inclusion in the second wave

	Model 1
Intercept	0.41 (0.22)***
During corona: How often financial support for others? Sometimes	0.76 (0.09)**
Most state institutions can be trusted in country? Is quite/ completely true	1.46 (0.10)***
Which best describes the area you live in? Big or middle size town	1.26 (0.08)**
Which emotions did you feel in relation others' behaviour? Grief	0.60 (0.15)***
Age category? <35 years	0.78 (0.09)**
I am someone who can forgive? <= 3 (7 points)	0.65 (0.11)***
Social differences in country are broadly fair? Is not/ a little true	0.68 (0.14)**
Worries about medical care? No worries	0.83 (0.08)*
What sensations do you associate with government? >= 7 (10 points)	0.77 (0.12)*
I am someone who can go out of himself, is sociable == 4 (7 points)	0.81 (0.10)*
How large is your circle of people you could count on? Rather/very large	1.26 (0.11)*
Self placement left/right scale? 5-8 center-right	1.21 (0.09)*
Worries about social cohesion? No worries	1.29 (0.11)*
Gay/lesbians free to live life as they wish? Disagree	1.49 (0.11)***
Education High	0.69 (0.09)***
Before Corona: How often contacted a politician? Often/ very often	1.72 (0.24)*
I am someone who has a vivid? imagination/ideas ==4 (7 points)	1.40 (0.12)**
I am someone who can forgive? ==4 (7 points)	0.56 (0.14)***
I am someone who is communicative/talkative? >= 5 (7 points)	0.78 (0.09)**
I am someone who is often worried? ==4 (7 points)	1.34 (0.10)**
I am someone who has a vivid imagination/ideas? >= 5 (7 points)	1.25 (0.11)*
None applies: home office/ children at home/ cared for relatives at home	1.20 (0.09)*
Berlin	1.50 (0.14)**
Hamburg	0.69 (0.18)*
What sensations do you associate with family? >= 7 (10 points)	1.22 (0.09)*
What sensations do you associate with government? 4, 5, 6 (10 points)	0.80 (0.10)*
How do you feel during corona? 4, 5 Determined	1.31 (0.10)**
Membership sports club	1.38 (0.13)*
No organisation membership	1.37 (0.11)**
Did your emotions target family and friends? Yes, pride	0.81 (0.09)*
Did your emotions target other groups? Yes, joy	0.65 (0.16)**
Support of relatives or friends	1.28 (0.10)*
Support of neighbours	0.83 (0.10)*
Support of unknown people	0.76 (0.14)*
How satisfied are you? >= 8 (11 points)	0.84 (0.09)*
Received support from neighbours	1.32 (0.11)*
Interested in politics? Not at all/ hardly interested	0.78 (0.09)**
McFadden Sq(R)	0.06
AIC	3858.74
BIC	4090.96
Num. obs.	3331

$^{***}p < 0.001$; $^{**}p < 0.01$; $^{*}p < 0.05$. Coefficients are included as odds ratios.

Table 2: Weights overview

	First wave Observed	Second wave Observed	First wave SocDem weight	Second wave SocDem (F1) + non-response weight
**Age**				
18-29	21.20	22.03	20.16	21.06
30-39	18.89	17.48	19.17	17.95
40-49	16.61	19.03	18.12	18.37
50-59	24.98	24.92	23.92	24.71
60-69	18.32	16.44	18.63	17.76
**Gender**				
Men	50.42	49.84	50.46	49.89
Women	49.58	50.05	49.54	49.97
**Education**				
High	36.94	31.64	29.37	28.59
Low/Middle	63.06	68.25	70.63	71.27
**Region**				
West	79.97	80.04	84.96	84.96
East	20.03	19.86	15.04	14.90

The values show percentage points.
